# Supplementary material for: Neuropsychological Assessments to Explore the Cognitive Impact of Cochlear Implants: A Scoping Review
Source: J Clin Med. 2025 Oct 27;14(21):7628. doi: 10.3390/jcm14217628 (PMC12608580; doi:10.3390/jcm14217628)
Supplement: Supplementary file 1 [file jcm-14-07628-s001.zip › Figure S1. Number of evaluations and proportion of cognitive gains after cochlear implantation by domain..pdf]

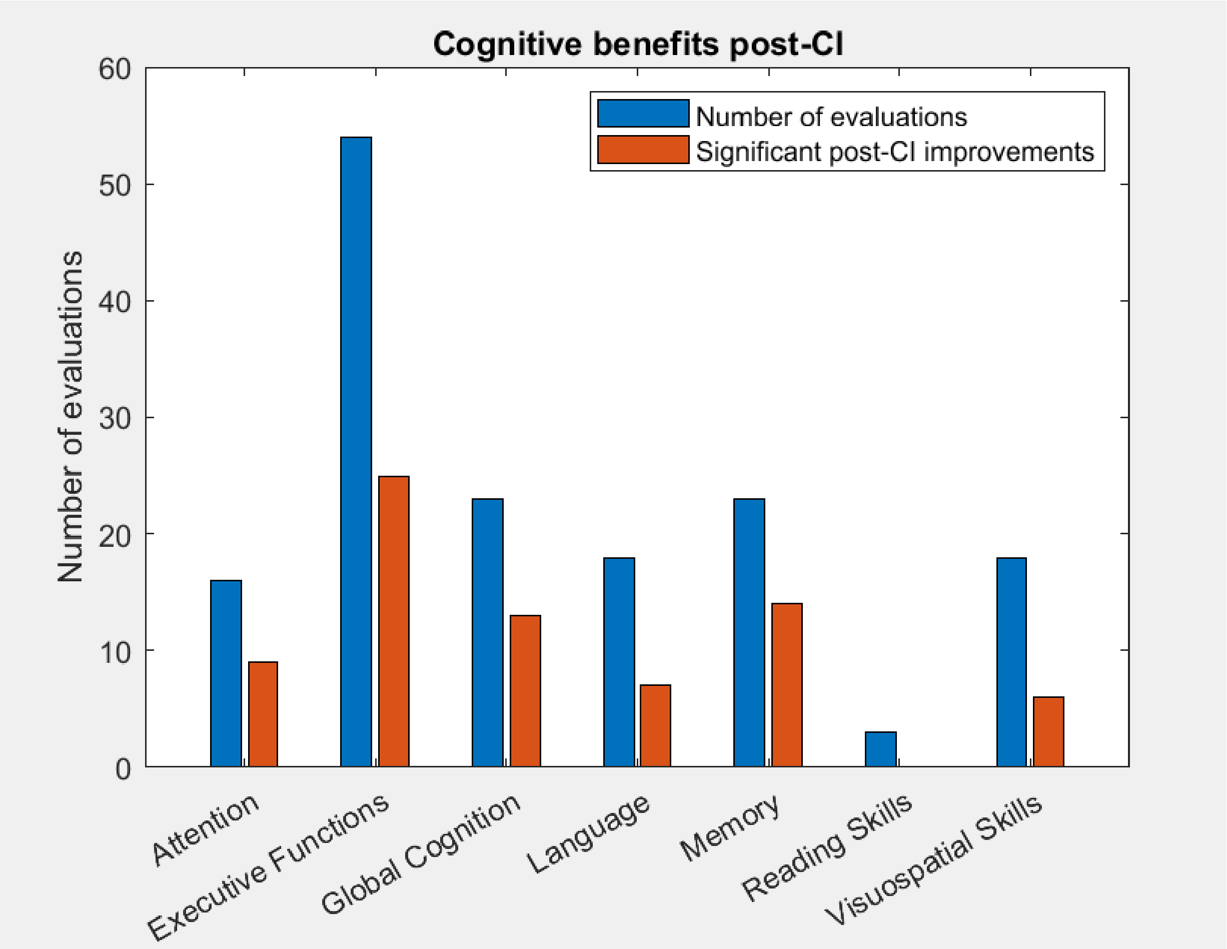

Figure S1. Number of evaluations and proportion of cognitive gains after cochlear implantation by domain.
