# Supplementary material for: Neuropsychological Assessments to Explore the Cognitive Impact of Cochlear Implants: A Scoping Review
Source: J Clin Med. 2025 Oct 27;14(21):7628. doi: 10.3390/jcm14217628 (PMC12608580; doi:10.3390/jcm14217628)
Supplement: Supplementary file 1 [file jcm-14-07628-s001.zip › Figure S2. Differences in cognitive status between CI users and other hearing profiles across domains.pdf]

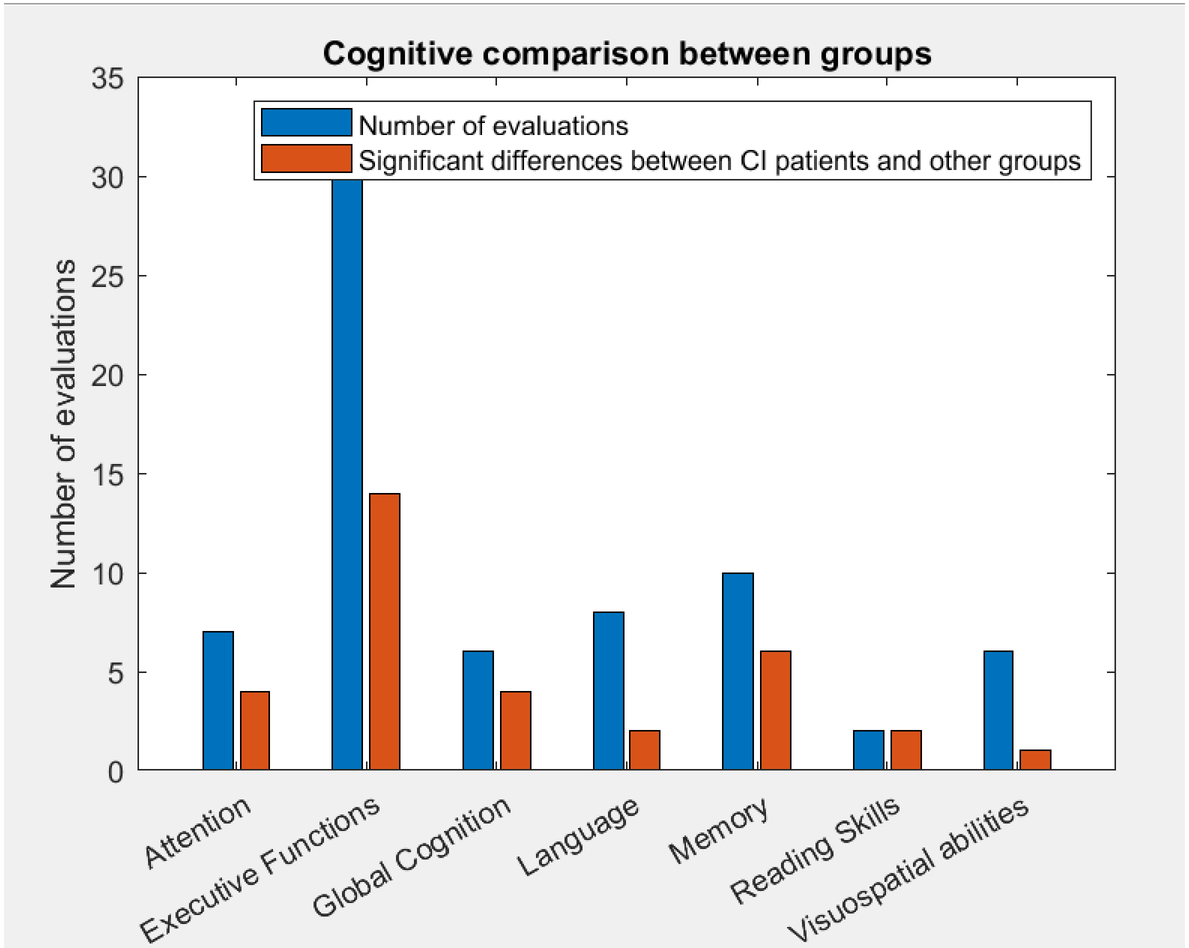


**Figure S2.** Differences in cognitive status between CI users and other hearing profiles across domains.
