# Supplementary material for: Neuropsychological Assessments to Explore the Cognitive Impact of Cochlear Implants: A Scoping Review
Source: J Clin Med. 2025 Oct 27;14(21):7628. doi: 10.3390/jcm14217628 (PMC12608580; doi:10.3390/jcm14217628)
Supplement: Supplementary file 1 [file jcm-14-07628-s001.zip › Supplementary Figures. Associations between cognition, audition and QoL.pdf]

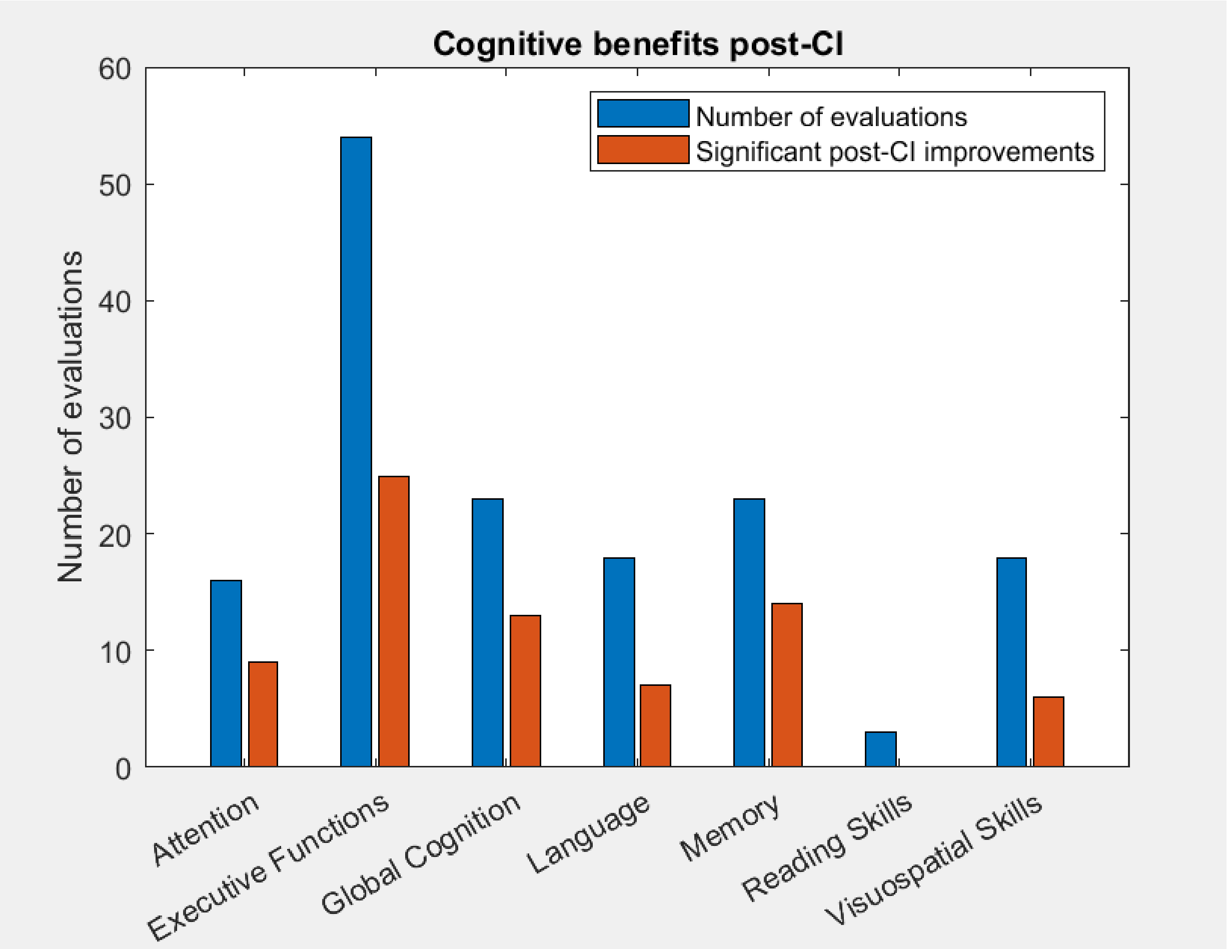

Supplementary Figure 1.1. Number of evaluations and proportion of cognitive gains after cochlear implantation by domain.

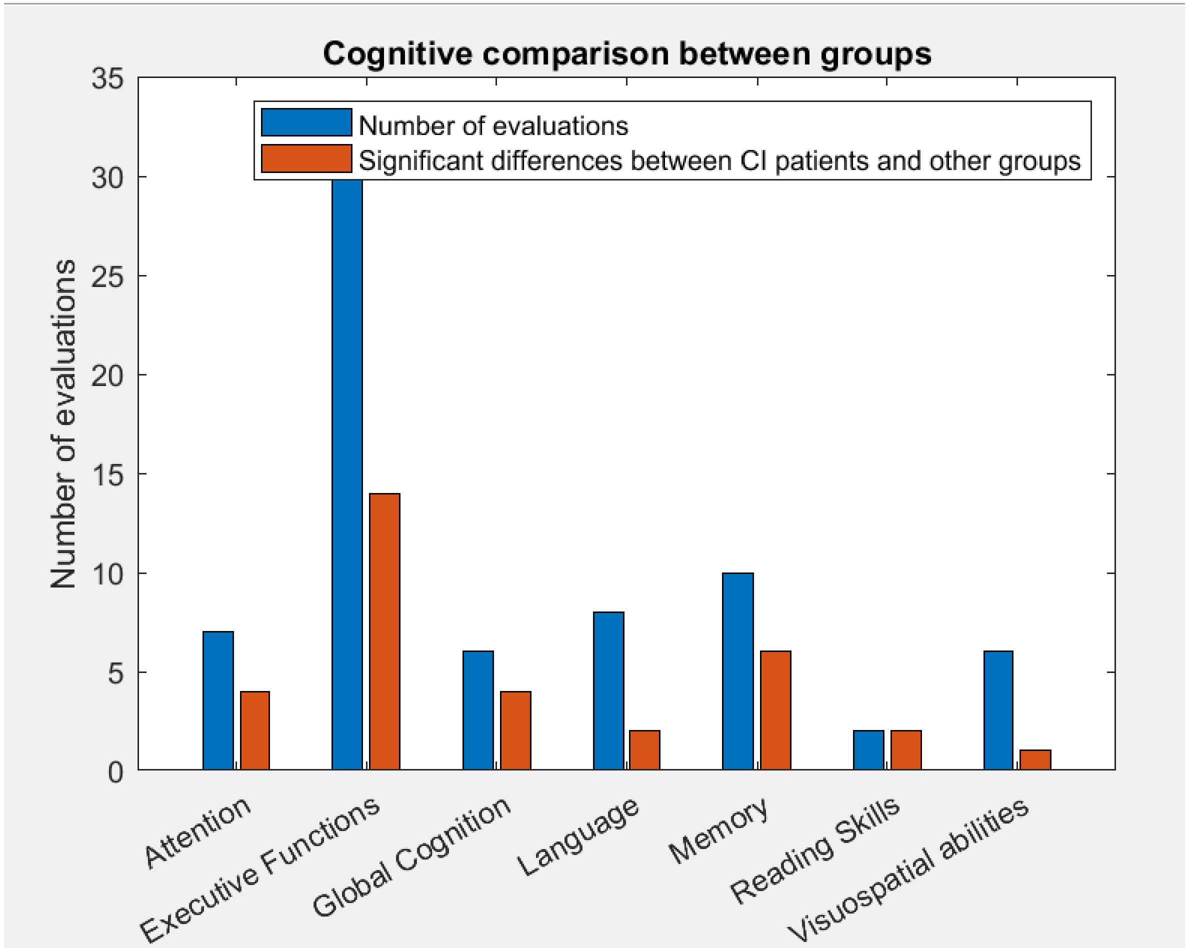

Supplementary Figure 1.2. Differences in cognitive status between CI users and other hearing profiles across domains.

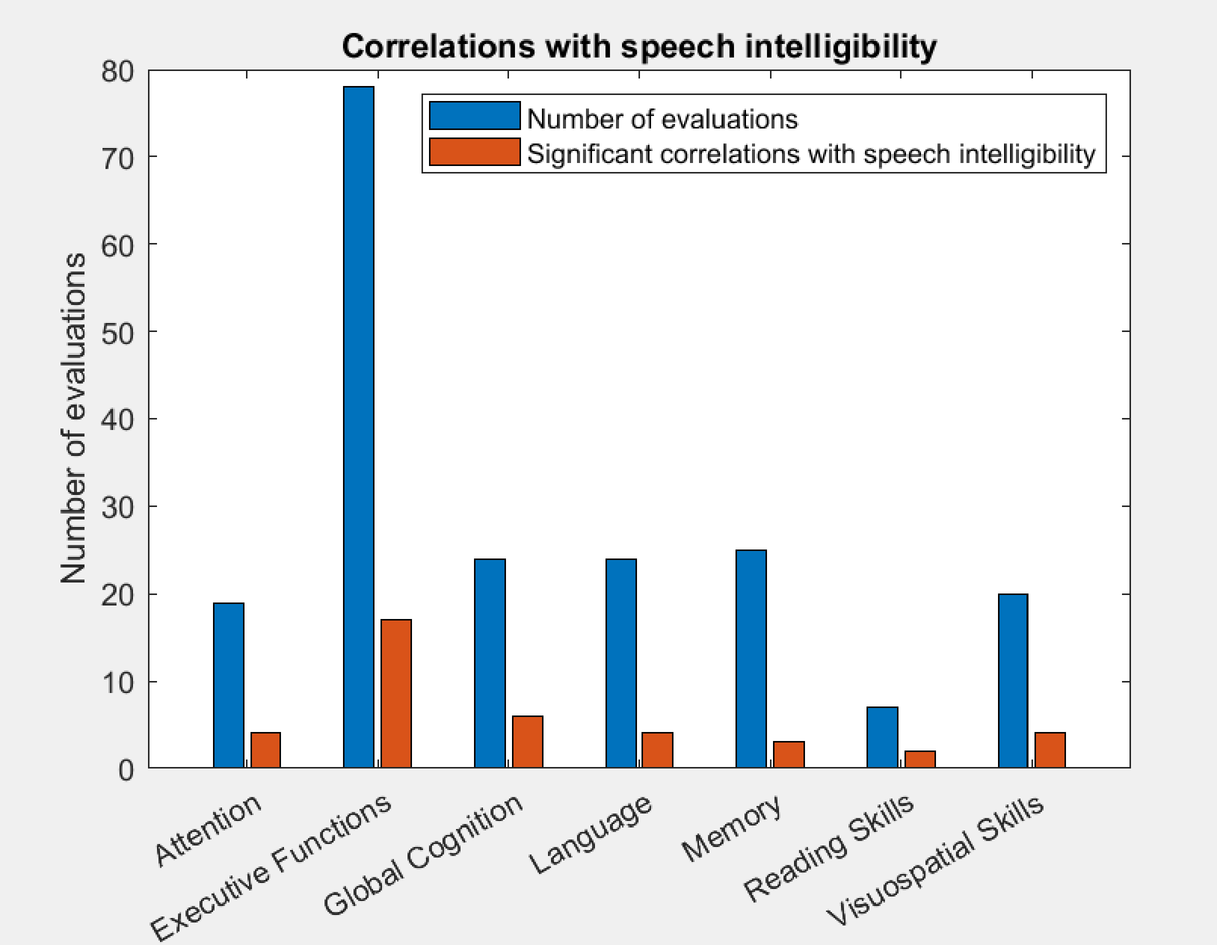

Supplementary Figure 1.3. Correlations between speech intelligibility and cognitive domain.

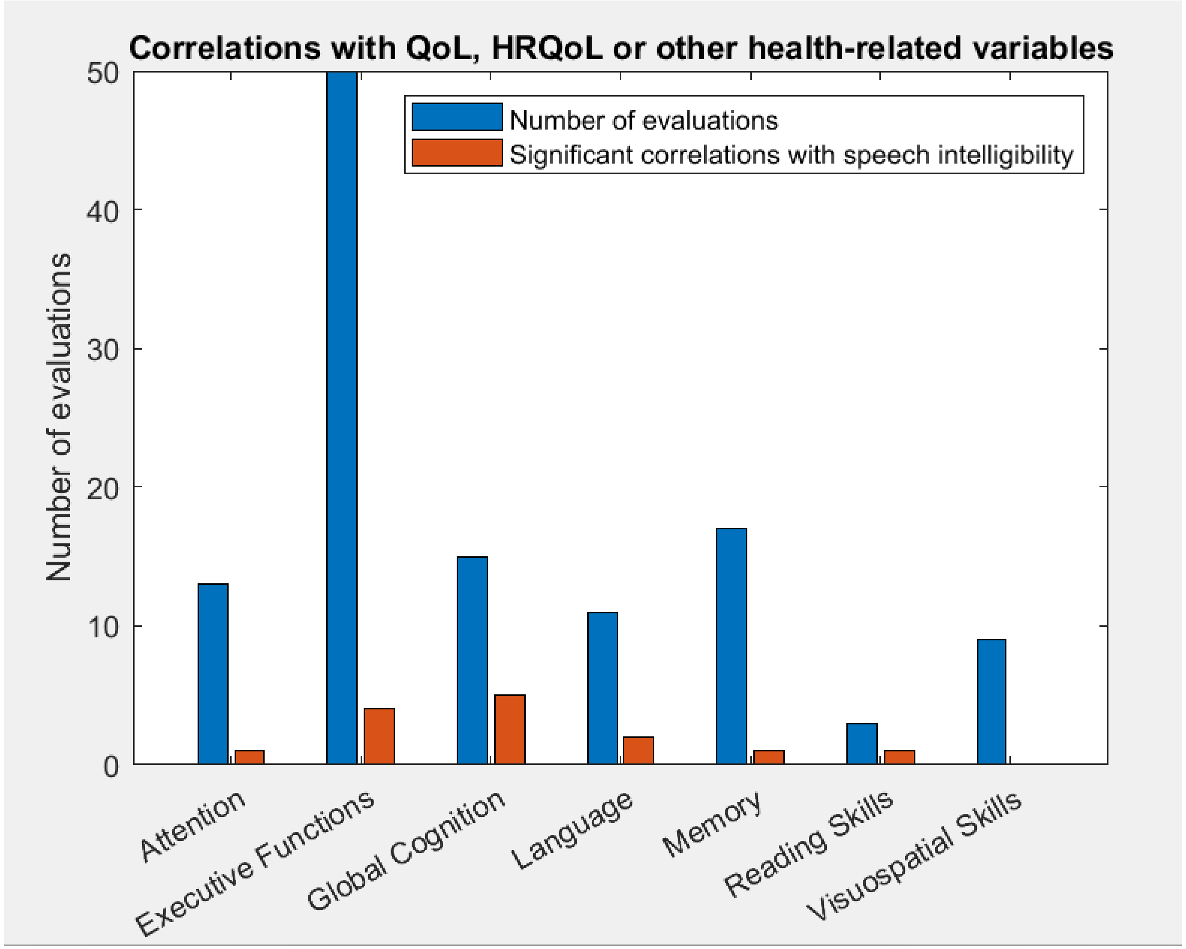

Supplementary Figure 1.4. Associations between cognition, quality of life (QoL), Hearing related QoL (HRQoL) and psychological well-being in CI users.
