# Supplementary material for: Neuropsychological Assessments to Explore the Cognitive Impact of Cochlear Implants: A Scoping Review
Source: J Clin Med. 2025 Oct 27;14(21):7628. doi: 10.3390/jcm14217628 (PMC12608580; doi:10.3390/jcm14217628)
Supplement: Supplementary file 1 [file jcm-14-07628-s001.zip › Table S1. Characteristics and Main Findings of Longitudinal Studies Assessing Cognitive Outcomes Before and After Cochlear Implantation in Adults With Hearing Loss.pdf]

Table S1. Characteristics and Main Findings of Longitudinal Studies Assessing Cognitive Outcomes Before and After Cochlear Implantation in Adults With Hearing Loss

Author(s), year<br><br>Design and Level of Evidence	Inclusion / Exclusion criteria	Participants<br>*(Mean age, ± SD and/or range)*	Time of Testing	Audiological assessment	Cognitive test (*domain measured*)	Other Variables and tests	Main cognitive findings	Principal conclusion
**Mosnier et al., 2015 [28]**<br><br>Prospective longitudinal study<br><br>2	≥ 65 years<br>Postlingual HL<br>CI candidacy<br>Bimodal users<br>No severe cognitive or medical conditions	CI candidates<br>n = 94<br>(72, [65-85])	Baseline (pre-CI)-<br>- 6-m post-CI<br>- 12-m post-CI	WRS in unilateral (CI/HA) and best-aided conditions, in quiet, noise (Fournier word lists)	*Global cognitive screening* (MMSE)<br>*Episodic memory* (5-Word test)<br>*Visuospatial abilities* (CDT)<br>*Language (*Verbal fluency)<br>*Attention and processing speed* (d2)<br>*Executive function and processing speed and*<br>(TMT A & B)	Educational level<br>QoL (NCIQ)<br>Depression (GDS-4)	At 12-m post-CI, 40% of patients had normal cognitive scores compared with 28% at baseline. The percentage of CI patients with three abnormal cognitive tests dropped from 20% at baseline to 5%. This improvement was significant as early as 6-m for global cognition, episodic memory, processing speed (d2), and executive function, and became significant at 12-m for processing speed (TMT-A) and the number of errors on the d2.<br>-Verbal fluency was the only predictor of improved speech perception in noise.<br>-After 12 months, 76% of patients showed no signs of depression (17% more than in baseline).	CI in older adults improves speech perception, cognitive function (especially in those with baseline impairment), depression and quality of life.
**Castiglione et al., 2016 [65]**<br><br>Longitudinal<br><br>2	≥ 65 years<br>moderate-to-severe HL<br>Group E: CI candidates<br>Group F: NH<br>No surgical complications or neurodegenerative conditions	Participants<br>Group E<br>n = 15<br>(71, [65–75])<br><br>Group F<br>n = 20<br>(70, [65–80])	Group E<br>-Baseline (pre-CI)<br>-1-y post-CI<br><br>Group F<br>Baseline	PTA<br>SDT and SRT with disyllabic/trisyllabic words and sentences in quiet<br>(N/A)	*Global cognitive screening (*MoCA)	Age at implantation<br>Depression (GDS)	Group E : Significant higher MoCA and lower GDS scores 1-y post-CI. MoCA scores were negatively associated with GDS (R^2=.7) and age at implantation (R^2 = .4); and positively mildly associated with PTA (R^2= .3) and SRT (R^2= .1).<br>A comparison of post-CI scores for Group E with NH suggests no significant differences in MoCA or GDS outcomes.	Positive effect of auditory rehabilitation on cognition and depression in CI candidates before and after implantation.
**Cosetti et al., 2016 [53]**<br><br>Longitudinal<br><br>2	≥ 65 years<br>Postlingual HL<br>CI candidacy<br>Bimodal users<br>Normal cochlear anatomy<br>Complete electrode array insertion<br>Normal cognition<br>No neurological condition	CI candidates<br>n = 7<br>(73.6 ±5.82, [67-81])	*Neurocognitive assessment:*<br>-Baseline (pre-CI)<br>-2-y post-CI<br>- 4.1-y post-CI<br>*Audiological assessment:*<br>- 1-y post-CI<br>- 2-y post-CI<br>- 3-y post-CI	WRS in quiet (CNCw)	*Premorbid intellectual function* (TOPF)<br>*Global cognition, language, abstract reasoning, Nonverbal abilities (*WASI: Vocabulary, Block Design, Similarities and Matrix Reasoning)<br>*Processing speed, cognitive flexibility (*TMT)<br>*Verbal Fluency* (Controlled Oral Word Association Tests)<br>*Visual naming* (BNT)<br>*Global cognition, short- and long-term memory, verbal fluency, attention, learning, mental flexibility, and motor-based processing speed* (RBANS)	Age at implantation<br>Education level<br>Marital status<br>Duration of HL<br>Etiology of HL<br>HA use<br>Comorbidities	Five cognitive tests (TOPF, WASI-VIQ, WASI-FSIQ, Vocabulary, BNT) showed significant and consistent associations with WRS in quiet 2 and 3-y post-CI. Similarities and Matrix Reasoning were predictive only in the bimodal group.	Speech perception and cognitive function were significantly interrelated over time, with five cognitive tests predicting better speech outcomes from 1-y to 3-y post-CI.
**Ambert-Dahan et al., 2017 [62]**<br><br>Prospective observational study<br><br>2	≥ 18 years<br>Severe-to-profound postlingual progressive SNHL<br>CI candidacy<br>No neurological, visual, or psychiatric illness	CI candidates<br>n= 18<br>(64 ± 3.5, [23-83]).	- Baseline (pre-CI)<br>- 12-m post-CI	Best-aided WRS in quiet (Fournier & Lafon) and SRS in quiet and noise (MBAA).	*Global cognition, short-term memory, spatiotemporal orientation* (CODEX)<br>*Global cognition, Visuospatial, naming, attention, verbal fluency, abstraction, memory* (MoCA)	Education level<br>Duration of HL	Among eight patients with pre-CI mild cognitive impairment, four improved to normal MoCA scores post-CI, with three of them also improving in the CODEX category. In those with normal baseline scores, cognition remained stable, with no significant declines. WRS and SRS significantly improved but were not associated with cognitive changes.	MoCA and CODEX are rapid and valuable screening tools for assessing cognitive function in hearing impaired subjects. Their use in routine clinical settings could enhance multidisciplinary monitoring of cognitive and auditory health in older adults.
**Jayakody et al., 2017 [60]**<br><br>Prospective patient-control study<br><br>2	CI candidacy	CI candidates (CIC)<br>n= 23<br>(69.04 ± 12.35)<br><br>CI recipients (CIR)<br>n = 16<br>(61.75 ± 15.62)	CIC:<br>-Baseline<br>- 6-m pre-CI<br>- 12-m pre-CI<br><br>CIR<br>- Baseline (pre-CI)<br>- 6 -m post-CI<br>- 12-m post-CI	PTA<br>WRS in quiet (CNCw)<br>SRS in noise (CUNY)	*Premorbid IQ* (NART-R)<br>*Global Cognition* CANTAB:<br>*Vision, comprehension, and hand movement* (MOT)<br>*Executive functioning and attentional set shifting* (AST)<br>*Recognition of complex visual patterns* (DMS)<br>*Episodic visuospatial memory, learning and association ability* (PAL)<br>*Immediate and delayed verbal memory* (VRM)<br>Reaction time-simple and complex (RTI)	Age<br>Etiology of HL<br>HA use<br>Duration of HL<br>Depression, Anxiety & Stress (DASS-21)	Group comparisons showed significant differences at 6-m for simple RTI and SWM. At 12-m, CIR outperformed CIC in AST, PAL, RTI and SWM. CIR also showed lower stress scores post-CI at 12-m.<br>Duration of HL was associated at baseline with higher AST latency and SWM errors. At 12-m post-CI, better CUNY scores correlated with faster AST responses, and CNC scores with better SWM strategy.	CI recipients outperformed CI candidates in several cognitive domains (simple reaction time, cognitive flexibility, paired-associate learning, working memory, and strategy use).

					Non-verbal, visuospatial working memory and strategy use (SWM)			
**Sonnet et al., 2017 [67]**<br>Prospective observational study<br>2	≥ 65 years<br>Postlingual severe-to-profound HL<br>CI candidacy<br>Normal cognition (MMSE ≥ 24 points)	CI candidates<br>n = 16<br>72.5 [65-80]	- Baseline (pre-CI)<br>- 6 -m post-CI<br>- 12-m post-CI	WRS in quiet (Lafon)	*Global cognitive screening* (MMSE)<br>*Executive functions* (ROCF,TMT)<br>*Memory* (5-Word test)<br>*Language denomination* (DO80)	Education level<br>HA use<br>Etiology of HL<br>QoL (WHOQOL, IADL & CIRS-G)	MMSE and other cognitive scores remained stable at 12-m post-CI, with improvement in executive function measured through TMT.	Global cognition remained stable 12-m post-CI, while executive functions showed improvement. CI also improves autonomy and QoL in the older adults.
**Claes et al., 2018 [77]**<br>Prospective, longitudinal cohort study<br>2	Postlingual HL<br>CI candidacy	CI candidates<br>n = 20<br>71.5 [54.8 - 84.8]	-Baseline (pre-CI)<br>- 6-m post-CI<br>- 12-m post-CI	WRS in quiet (NVA list)<br>SRS in noise (LIST)	*Immediate Memory, Visuospatial Constructional, Language, Attention & Delayed Memory* (RBANS-H)	HA use<br>HRQoL (NCIQ, HISQUI19 SSQ12)<br>Anxiety & Depression (HADS)	Significant improvement in RBANS-H total score after 12-m, mainly due to gains in Immediate Memory, and Delayed Memory.<br>NCIQ, SSQ12, and HISQUI19 all improved significantly at 6-m and remained stable at 12 m.<br>HADS scores showed significant reductions in anxiety and depression at 6-m, with reduced benefit at 12-m.	After 12-m of CI use, overall cognition improved significantly. Positive effects on speech perception and HRQoL,QoL and mood disorder were also observed.
**Mosnier et al., 2018 [68]**<br>Prospective longitudinal study<br>2	≥ 65 years<br>Postlingual severe-to-profound HL<br>CI candidacy<br>Normal cognition<br>No severe medical disorder	CI candidates<br>n = 70<br>(72 ±5.1, 65-85)	- Baseline (pre-CI)<br>- 1y post-CI<br>- 7y post-CI	WRS in unilateral (CI/HA) and best-aided conditions, in quiet, noise (Fournier word lists)	*Global Cognitive Screening* (MMSE)<br>*Verbal and semantic memory* (5-Word test)<br>*Visuospatial, long-term attention, memory, auditory processing, and motor skills* (CDT)<br>*Language: Phonetic and semantic fluency* (Verbal fluency test)<br>*Attention* (d2)<br>*Executive function and processing speed* (TMT A&B)	Living status<br>Age at implantation<br>Duration of HL<br>HA use<br>HRQoL (NCIQ)	At 1-y post-CI, global cognition, executive function and attention declined significantly, while memory (5-word) and verbal fluency remained stable. No significant correlations were found between cognitive changes and WRS.<br><br>After 7-y, only 6% progressed to dementia, 61% remained stable, and 32% returned to normal cognition. Among those with normal baseline cognition, 32% developed MCI. Overall, there was no significant change in the number of participants with cognitive impairment from baseline (45%) to 7-y (48%).	CI may help stabilize or improve cognitive function in older adults with MCI, with a low rate of progression to dementia over 7-y. Hearing rehabilitation may offer neurocognitive benefits even in those with baseline impairment.
**Völter et al., 2018 [56]**<br>Prospective, longitudinal cohort study<br>2	≥ 50 years<br>severe-to-profound HL<br>CI candidacy<br>No neurologic or psychiatric disorders<br>No uncorrected vision	CI candidates<br>n = 60<br>(65.8 ± 8.9)	-Baseline (pre-CI)<br>- 6-m post-CI<br>- 12-m post-CI	PTA<br>WRS in quiet (Freiburg)<br>SRS in noise (OLSA)	*ALA cog:*<br>*Attention (M3 test)*<br>*Immediate and delayed memory (Recall and delayed recall task)*<br>*Working memory (0-back, 2-back and Operation Span)*<br>*Inhibition (Flanker test)*<br>*Executive function and processing speed (TMT A&B)*<br>*Verbal fluency and executive control (Chicago Word Fluency Test)*	Duration of HL<br>HA use<br>HRQoL (NCIQ)<br>QoL (WHOQOL)	At 6-m, speech perception, QoL, and cognitive abilities improved significantly. The greatest gains were in attention, inhibition, working memory (n-back& operation span) and delayed recall. Long-term memory improved after 12-m. Most domains remained stable after 6-m, except for working memory (operation span), which continued to improve up to 12-m.<br>No associations were observed with HL duration, speech outcomes, or QoL.	CI enhances speech perception, QoL, and cognitive function in adults over 50, with these improvements occurring independently.
**Anzivino et al., 2019 [69]**<br>Prospective, multicentric longitudinal study<br>2	≥ 60 years<br>severe-to-profound HL<br>Normal cognition (MMSE ≥ 24 points)<br>≥ 8 years of education<br>Motivated for auditory rehabilitation<br>No neurological disease or impairment affecting rehabilitation	CI candidates<br>n = 25<br>(66.4 ± 5.8)<br><br>Hearing Aid Candidates<br>n = 19<br>(74.92 ± 5.4)	-Baseline (pre-CI/HA)<br>- 6-m post- CI/HA<br>-12-m post-CI	PTA (unaided, best-aided and residual hearing)<br>WRS in quiet (Turrini).<br>SRS in quiet and noise (Burdo/Orsi)	*Global Cognitive screening* (MMSE)<br>*Episodic memory* (RAVLT)<br>*Working Memory* (ROCF, Digit Span & Corsi Span)<br>*Attention (*MFTC & TMT A& B)<br>*Executive Functions* (SCWT)<br>*Semantic memory* (Phonological and Categorical Verbal Fluency)	Education level<br>HA use<br>Etiology of HL<br>QoL (SF-36, GBI & GHSI)	At 6-ms, both CI and HA groups showed significant gains in MMSE and RAVLT, with no difference between groups. At 12-m, CI users maintained cognitive gains, with improved executive function (SCWT).<br>Significant correlation of RAVLT and MMSE scores with SRS in noise for SNRs of +10 and 0 dB.	Hearing rehabilitation with CI or HA improves memory and global cognition within 6-m. CI users may show further executive function gains by 12-m. Auditory rehabilitation may support cognitive resource reallocation in older adults.

**Sarant et al., 2019 [63]**<br><br>Prospective longitudinal study<br><br>2	≥ 60 years<br>severe-to-profound HL<br>CI candidacy<br>Normal cognition<br>(MMSE ≥ 24 points)	CI candidates<br>n = 59<br>(72.3 ± 6.8)	-Baseline (pre-CI)<br>-18-m post-CI.	PTA<br>WRS in quiet (CVCw)<br>SRS in noise (BKB)	*Global Cognitive screening* (MMSE)<br>Cogstate Brief Battery:<br>*Executive Function* (GMLT)<br>*Psychomotor function* (Detection Task)<br>*Visual learning* (OCLT)<br>*Visual attention* (Identification Task)<br>*Working memory* (One Back Task)	Education Level<br>HA use<br>QoL (HUI-3 & IPAQ)<br>HRQoL (APHAB, Bayer)<br>Anxiety and depression (HADS)<br>Loneliness (LSNS, Loneliness Scale)	At baseline, higher PTA in the better ear predicted poorer executive function (GMLT). Higher education was associated with better cognitive performance. At 18-m, executive function (GMLT) improved significantly in males without tertiary education, with no significant changes in other cognitive domains.<br>Loneliness, social isolation, depression and anxiety showed non-significant improvements.	CI significantly improved speech perception and QoL, and helped preserve cognitive function. Executive function improved notably in less educated males, suggesting CI may support cognitive stability in high-risk older adults.
**Sorrentino et al., 2020 [70]**<br><br>Longitudinal and cross-sectional cohort study<br><br>2	≥ 65 years or ≤50 years<br>Profound HL<br>Postlingual HL<br>Prior HA use ≥ 1 year<br>CI candidacy<br>Regular fitting and speech therapy<br>NH control matched on age, comorbidities, education	Older CI group<br>n = 25<br>71 [67 – 75]<br><br>Younger CI group<br>n =19<br>39 [28-43]<br><br>NH<br>n = 25 [> 65]	-Baseline (pre-CI),<br>-12-m post-CI post-activation, -Final follow-up visit (mean 42-m).	PTA<br>WRS in quiet (Test Abilità Uditive Varese)	*Global cognitive screening* (MMSE)	Education level<br>HA use<br>QoL (GBI).	MMSE scores in older CI users were comparable to NH peers.<br>MMSE correlated positively with WRS (r = .44, p = .01), time since implantation (r = .38, p = .03), and GBI physical health (r = 0.41, p = .02).	CI in older adults improves WRS in quiet and QoL to levels similar to younger CI recipients and may help preserve cognitive function comparable to NH control matched-age adults.
**Zhan et al., 2020 [84]**<br><br>Prospective cohort study<br><br>2	≥ 18 years<br>severe-to-profound HL<br>CI candidacy<br>Normal cognition<br>(MMSE ≥ 24)<br>Word reading<br>(WRAT ≥70)<br>No significant visual impairments	CI candidates<br>n = 19<br>(67.8 ±9.7)<br><br>NH<br>n = 43	-Baseline (pre-CI)<br>-6-m post-CI.	PTA<br>WRS in quiet (CID)<br>SRS in quiet and in noise (AzBio, Harvard, & PRESTO)	*Working memory* (Visual digit span, visual object span, and visual symbol span)<br>*Processing speed* (SCWT)<br>*Nonverbal reasoning* (Raven's)	Etiology of HL<br>Duration of HL<br>HA use<br>Socioeconomic status	Demonstrated cognitive improvement after CI in WM and processing speed SCWT. Greater improvement was seen in participants with lower baseline scores. No change in Raven's matrices.<br>Preoperative WM scores predicted WRS and SRS both in quiet and in noise at 6 months, while SCWT incongruent scores specifically predicted SRS in quiet. Duration of hearing loss correlated negatively with SRS.	CI improves executive function, especially in individuals with lower baseline cognitive performance. Preoperative cognitive ability, particularly WM and inhibition, predicts speech recognition outcomes.
**Huber et al., 2021 [61]**<br><br>Prospective cohort study<br><br>2	60–80 years<br>Bilateral severe-to-profound HL<br>CI candidacy<br>German-speaking<br>Normal Cognition<br>(MMSE ≥ 24)<br>No significant visual impairment<br>No anticholinergic medication	CI candidates<br>n = 29<br>(70.62 ± 4.67)<br><br>NH<br>n = 29<br>(68.45 ±4.88)	-Baseline (pre-CI)<br>-12-m post-CI	PTA<br>WRS in quiet (FMT)<br>SRS in quiet and noise (HSM).	*Global Cognition and visuospatial skills* (CDT)<br>CERAD:<br>*Verbal episodic memory immediate and delayed recall* (WLLR)<br>*Figural episodic memory immediate and delayed recall* (CPR)<br>*Executive function, attention, processing speed* (SCWT)<br>*Executive function, cognitive flexibility* (TMT A &B)	Education Level<br>Etiology of HL<br>Duration of HL<br>HA use<br>Smoking<br>Hypertension<br>Diabetes<br>Auditory training	Considerably improvement in global cognition (CDT) post-CI, reaching similar scores than NH controls.<br>No significant improvements in verbal and figural episodic memory, or executive function. CI still showed lower performance than controls in WLLR, CPR, delayed recall CPR, SCWT and TMT-B after 12-m. CDT improvement correlated with WRS and SRS in quiet at 3-m, whereas not at 12-m.	CI improved global cognition in older adults to a level comparable with age-matched NH controls, but deficits in verbal memory (WLLR) and executive function SCWT and TMT-B) remained. CI may partially counteract hearing loss-related cognitive decline.
**Knopke et al., 2021 [82]**<br><br>Prospective, non-interventional, and longitudinal study.<br><br>2	≥ 70 years<br>Bilateral severe HL<br>Postlingual HL<br>No retrocochlear disorders or anatomical limitations<br>No dementia or MCI<br>No severe visual impairment	CI candidates<br>n = 21<br>(77.1 ± 5.5)	-Baseline (pre-CI)<br>-12-m post-CI	PTA<br>WRS in quiet (FMT)<br>SRS in noise (OLSA).	WAIS-IV:<br>*Working Memory* (Digit span and Arithmetic)<br>*Processing Speed*<br>(Symbol Search and Digit Symbol Coding)	Duration HL<br>HA use<br>Depression (ADS-L)<br>QoL (OI) .<br>Speech therapy sessions	Significant improvement in Working Memory Index and Processing Speed Index at 12-m, with no significant changes in individual subtests.<br>No correlation was found between cognitive scores and speech perception or depression scale.	CI in adults over 70 leads to significant gains in working memory and processing speed, supporting cognitive benefit from auditory rehabilitation.
**Mertens et al., 2021 [78]**<br><br>Prospective, longitudinal, controlled, multicenter study<br><br>2	≥ 55 years<br>Bilateral severe-to-profound HL<br>Postlingual HL<br>Unilateral CI candidates<br>CI use ≥ 10 hours/day	CI candidates<br>n = 24<br>(72 ±7)<br><br>Control group (patients with contraindication for CI)<br>n = 24<br>(73 ± 9)	-Baseline (pre-CI)<br>-14-m post-CI	PTA<br>Residual hearing in the best ear<br>Speech perception in quiet and in noise (clinical routine local test batteries)	*Immediate Memory, Visuospatial Constructional, Language, Attention & Delayed Memory* (RBANS-H)	Education level<br>Etiology of HL<br>Duration of HL<br>HA use<br>Residual hearing<br>Working status<br>Personality (DS14)<br>Anxiety and Depression (HADS)<br>HRQoL (HISQUI-19, SSQ-12)<br>Tinnitus	RBANS-H total score notably increases in CI users 14-m post-CI, with a trend towards greater gain than controls. The improvement in attention was considerably greater in the CI group than in controls. Gains in Immediate and Delayed Memory in CI users became nonsignificant after correcting for practice effects.<br>CI users showed reduced negative affectivity and social inhibition.	CI in older adults notably increase cognition particularly attention and reduces negative personality traits. However, CI users did not fully match cognitive levels of NH peers after 1-y of implantation, suggesting the need for extended or additional cognitive rehabilitation.
**Vasil et al., 2021 [66]**	55–85 years<br>Severe-to-profound HL	CI candidates<br>n = 77<br>(72.4 ± 6.4)	-Baseline (pre-CI)<br>-6-m post-CI	WRS in quiet (CNCw)	*Global Cognitive screening* (MoCA)	Duration of HL<br>HA use	The MoCA scores markedly enhanced 6-m post-CI, primarily driven by gains in the Delayed Recall	CI led to gains in MoCA performance with more participants reaching the cutoff threshold for

Prospective cohort study<br>2	Postlingual HL<br>CI candidacy<br>SRS < 60% (AzBio)<br>< 20 years severe HL<br>No visual or medical conditions affecting cognition						domain. MoCA scores were significantly predicted by post-CI WRS in quiet.	normal cognition, mainly due to better performance on auditory-dependent tasks. Cognitive screening outcomes post-CI were related to speech perception ability.
**Völter et al., 2021 [57]**<br>Prospective longitudinal study<br>2	≥ 50 years<br>Bilateral severe-to-profound HL<br>Postlingual HL<br>4-PTA ≥ 61 dB on a better ear.<br>No global cognitive impairment (MWI-B)<br>No CNS disease<br>No anticholinergic medication<br>No severe depression (GDS-15)<br>No uncorrected vision.	CI candidates<br>n = 71<br>(66.3 ± 9.2)<br>NH<br>n = 105<br>(65.96 ± 9.38)	- Baseline (pre-CI)<br>- 12-m post-CI	PTA<br>WRS in quiet (Freiburg)<br>SRS in noise (OLSA)	ALA cog:<br>*Attention* (M3 test)<br>*Immediate and delayed memory* (Recall and delayed recall task)<br>*Working memory* (0-back, 2-back and Operation Span)<br>*Inhibition* (Flanker test)<br>*Processing speed and executive function (TMT A&B)*<br>*Verbal fluency and executive control* (Chicago Word Fluency Test)	Education level<br>Etiology of HL<br>Duration of HL<br>HA use<br>Depression (GDS-15)	Substantial gains were observed at 12-m in M3, recall, delayed recall, 2-back, OSPAN, iFlanker, and verbal fluency. Although no change was detected in 0-back, cFlanker, TMT A/B. Greater cognitive gains were present in patients with lower baseline scores.<br>A significant correlation appeared in post-CI attention (M3) and WRS in quiet as well as SRS in noise. Also noted in enhance working memory (0- and 2-back) and better SRS in noise.	CI improves multiple cognitive domains, especially attention, working memory, and verbal fluency. Leading to recipients achieving cognitive performance similar to age-matched NH peers after 12-m.
**Calvino et al., 2022 [79]**<br>Prospective longitudinal cohort study<br>2	≥ 12 months CI use<br>Bilateral severe-to-profound HL<br>Postlingual HL<br>Normal cognition<br>No severe depression<br>No CNS disease<br>No psychiatric disorders	CI candidates<br>< 60 years<br>n = 28<br>(48.7 ± 8.3)<br>≥ 60 years<br>n = 35<br>(70.5 ± 6.2)	- Baseline (pre-CI)<br>- 12-m post-CI	PTA<br>WRS in quiet and noise (Cárdenas & Marrero)<br>SRS in quiet and noise (Davis & Silverman)	*Immediate Memory, Visuospatial Constructional, Language, Attention & Delayed Memory* (RBANS-H)	Hl aetiology<br>Duration of HL<br>HA use<br>QoL (GBI)<br>HRQoL (NCIQ, HISQUI19<br>SSQ12)<br>Anxiety & Depression (HADS)	RBANS-H total score improved significantly in both age groups. The <60y group enhances Immediate and Delayed Memory. As the ≥60y group boost in Delayed Memory, Language and Visuospatial domains.<br>Among participant ≥60y, age negatively correlated with post-CI RBANS-H total and domain scores. RBANS-H improvement correlated with both post-CI speech perception and NCIQ self-esteem.	CI led to cognitive improvement, enhanced speech perception, and better QoL in both age groups. After 12-m of CI use, older adults benefited the most in cognitive domains. Although younger adults outperformed older adults, at post-CI both groups reached similar cognitive levels.
**Gurgel et al., 2022 [55]**<br>Prospective, interventional cohort study<br>2	≥ 65 years<br>CI candidacy<br>No contraindication for surgery<br>No vision impairment or color blindness	CI candidates<br>n = 37<br>79.4 (7.4)	- Baseline (pre-CI)<br>- 6-m post-CI<br>- 14-m post-CI	PTA<br>SRS in quiet and in noise (AzBio & HINT)	*Global Cognitive screening* (MMSE)<br>*Attention and working memory* (Digit Span Test)<br>*Inhibition* (SCWT)<br>*Verbal memory and learning* (HVLT-R)<br>*Executive function and verbal reasoning* (Hayling)<br>*Attention, short-term and working memory* (Spatial Span)<br>*Attention and concentration* (d2)<br>*Verbal learning and memory* (BVMT)<br>*Attention, processing speed, and mental flexibility* (TMT A & B)	Education level<br>Visual impairment<br>Age at implantation<br>HA use<br>Depression (GDS)	The entire cohort showed a notable increase in Digit Span, HVLT-R total and delayed, BVMT-R, d2 concentration, and Trail B score. The subgroup with pre-CI cognitive impairment showed greater gain, especially in HVLT-R total T-score, SCWT T-score and BVMT-R delayed raw score.	CI leads to gain in cognitive capacity, particularly verbal memory, attention, and executive function. Benefits were most pronounced in those with preoperative cognitive impairment, who achieved post-CI scores comparable to those with NH.
**Herzog et al., 2022 [71]**<br>Prospective cohort study<br>2	≥ 65 years<br>Bilateral SNHL<br>Postlingual HL<br>CI candidacy<br>Normal cognition (MMSE ≥ 24)	CI candidate<br>n = 53<br>75 [65-97]	- Baseline (pre-CI)<br>- 1-y post-CI<br>- 4+-y post-CI	PTA<br>WRS in quiet (CNCw)<br>SRS in quiet and in noise (AzBio & HINT)	*Global Cognitive screening* (MMSE)	Duration of HL<br>Severity of HL<br>Duration of HL<br>Cardiovascular disease<br>Stroke<br>Diabetes<br>MRI vessel changes, atrophy	Long-term MMSE decline did not differ between patients with normal vs mildly impaired cognition at baseline. Overall MMSE declined by 1.3 points over 4+-y; rate ~0.18 points/year, within expected aging range. Of 53 patients, 8 an MMSE ≤ 24; 6 of these 8 patients had MRI abnormalities at baseline, although these were not linked to MMSE decline.	Post-CI speech perception improvement was sustained over 4+-y regardless of pre-CI cognitive status. Cognitive decline rates were similar between groups, suggesting that CI may help maintain cognitive function in older adults.
**Ohta et al., 2022 [72]**<br>Prospective longitudinal cohort study<br>2	≥ 65 years<br>Bilateral profound or severe bilateral SNHL<br>PTA ≥ 70 dB<br>Speech intelligibility ≤ 50% with HA<br>No inner ear malformations<br>No contraindications for general anesthesia<br>No psychiatric illness	CI candidates<br>n = 21<br>69 [65-80]	- Baseline (pre-CI)<br>- 1-y post-CI<br>- 2-y post-CI	PTA<br>WRS in quiet (monosyllabic & CI2004)<br>SRS in quiet and noise (CI2004)	*Global Cognitive screening* (MMSE)	HA use<br>Etiology of HL<br>Duration of HL<br>HRQoL (NCIQ)<br>Depression (SDS)	MMSE significantly increase from baseline to 1-y post-CI. Although there was a slight non-significant decline at 2-y, and MMSE at this time point significantly correlated with the NCIQ speech production subdomain (r = .47, p = .031).	CI in older adults boost cognitive function within the first year, wich is associated with better speech production. Cognitive benefits may stabilize or slightly decline after one year but remain above baseline.
**Völter et al., 2022 [58]**<br>Prospective, longitudinal study	≥ 50 years<br>Bilateral severe-to-profound HL<br>Postlingual HL<br>4-PTA ≥ 61 dB	CI candidates<br>n = 71<br>(66.03 ± 9.15)	- Baseline (pre-CI)<br>- 6-m post-CI<br>- 12-m post-CI<br>- 65-m post-CI	PTA<br>WRS in quiet (FMT)	ALA cog:<br>*Attention* (M3 test)<br>*Immediate and delayed memory* (Recall and delayed recall task)	Education level<br>Etiology of HL<br>Duration of HL<br>HA use<br>HRQoL (NCIQ)	Gains in attention, working memory (Operation Span) and inhibition are noticed at 6-m from baseline. Improvements in memory recall and verbal fluency were noted at 12-m, and working memory (2-back) at 24-m. CRIq was positively	CI significantly enhanced attention, inhibition, and working memory by 6-m, while memory and verbal fluency improved within 12-m, particularly in individuals with low

2	No global cognitive impairment (MWI-B)<br>No CNS disease<br>No anticholinergic medication<br>No uncorrected vision				*Working memory* (0-back, 2-back and Operation Span)<br>*Inhibition* (Flanker test)<br>*Processing speed and executive function (TMT A&B)*<br>*Verbal fluency* (Verbal fluency task)	Cognitive Reserve (CRIq)<br>Depression (GDS-15)	correlated with cognition pre- and post-CI. However, participants with lower CRIq showed greater gain in attention.<br>Even though no correlation found between cognitive improvement and WRS or QoL	pre-CI cognitive reserve. Cognitive gains remained stable throughout the 24-m but were not linked to WRS or QoL.
**Zucca et al., 2022 [73]**<br><br>Prospective study<br><br>2	≥ 50 years<br>Postlingual HL<br>CI candidacy<br>No severe visual impairment	CI candidates<br>n = 21<br>(65 ± 8)	- Baseline (pre-CI)<br>- 12-m post-CI	PTA<br>WRS in quiet (CVC)	*Global Cognitive screening* (MMSE)<br>*Working Memory* (CDT & Digit-Span)<br>*Visuospatial memory* (Corsi Block-tapping Test)<br>*Phonemic and semantic fluency* (Verbal Fluency Test)<br>*Attention and Processing Speed* (TMT A&B)<br>*Memory* (RAVLT)	Education level<br>Etiology of HL<br>Duration of HL<br>HA use	No significant baseline cognitive differences between high vs. low speech performers (cutoff < 80%). Trend for better semantic verbal fluency in high performers. TMT-A scores were the only significant cognitive predictor of WRS in quiet at 12-m ($\beta = -0.486$, $p = .035$).	Cognitive processing speed (TMT-A) and younger age are predictors of better WRS in quiet post-CI.
**Andries et al., 2023 [80]**<br><br>Prospective, longitudinal cohort study<br>2	≥ 55 years<br>Bilateral severe-to-profound HL<br>Postlingual HL<br>CI candidacy<br>(RBANS-H total score ≤ 16th percentile)	CI candidate<br>n = 21<br>(72 ± 9)	- Baseline (pre-CI)<br>- 12-m post-CI	PTA<br>SRS in noise (LIST)	*Immediate Memory, Visuospatial Constructional, Language, Attention & Delayed Memory* (RBANS-H A & B)	Education level<br>Etiology of HL<br>HA use<br>Anxiety & Depression (HADS)	RBANS-H total percentile improved significantly at 12-m post-CI. As well as immediate and delayed memory improved. These were not associated with education, sex, HADS, or RBANS-H A & B versions. Better SRS in noise was significantly associated with greater cognitive improvement ($r = -0.48$, 95% CI: –0.69 to –0.19).	CI significantly improves global and memory-related cognitive function in older adults at risk for MCI. Positive changes in speech perception are associated with cognitive gains.
**Baranger et al., 2023 [54]**<br><br>Prospective, observational study<br><br>3	> 60 years<br>Normal cognition (MMSE ≥ 24)<br>Education and cultural level ≥ 8 years<br>No neurological or cognitive disorder<br>No visual impairment	CI candidates<br>n = 43<br>(55.7, 20-85)	- Baseline (pre-CI)<br>- 3-m post-CI	PTA	*Phonemic and semantic fluencies* (Cardebat's fluencies)	Education level<br>Etiology of HL	At baseline, phonemic fluency scored significantly higher than semantic fluency, and they were positively correlated. Congenital deafness was linked to better semantic fluency than acquired deafness. After 3-m of CI, patients showed significant improvement in phonemic fluency but not in semantic fluency. Correlation between semantic or phonemic fluency gains and PTA was not found.	As soon as 3-m after CI, users showed a significant improvement in phonemic fluency independently of the etiology of HL. This adds proof to the usefulness of CI in cognitive stimulation in the elderly. CI led to early gains in phonemic verbal fluency, indicating improved access to sound-based language representations.
**Haeussler et al., 2023 [83]**<br><br>Prospective, longitudinal study<br><br>2	> 65 years<br>Bilateral severe HL<br>CI candidacy	CI candidates<br>n = 33<br>(75.5 ± 4.9)	- Baseline (pre-CI)<br>- 1-y post-CI<br>- 2-y post-CI	PTA<br>WRS in quiet (FMT)	WAIS-IV:<br>*Working Memory* (Digit span and Arithmetic)<br>*Processing Speed* (Symbol Search and Digit symbol Coding)	Education level<br>HA use<br>Etiology of HL<br>Duration of HL<br>QoL (OI)<br>Stress (PSQ)<br>Depression (ADS-L)	The Working Memory Index showed significant improvement 2-y after cochlear implantation, while the Processing Speed Index improved significantly at both 1-y and 2-y post-CI. Working Memory Index correlated positively with years of education at all time points.	CI in older adults resulted in sustained improvement in working memory and processing speed over a 2-year period, with cognitive gains not directly related WRS in quiet. Education level was positively associated with working memory.
**Völter et al., 2023 [59]**<br><br>Prospective, longitudinal cohort study<br><br>2	≥ 50 years<br>Bilateral HL<br>CI candidacy<br>No visual impairment	CI candidates<br>n = 75<br>(65.4 ± 9.19)<br><br>Health and Retirement Study (HRS) and the English Longitudinal Study of Aging (ELSA)<br>n = 8,077<br>(65.4 ± 9.19)	- Baseline (pre-CI)<br>- 12-m post-CI<br>- 24-m post-CI<br>- 5-y post-CI	PTA<br>WRS in quiet (FMT)	ALAcog:<br>*Verbal episodic memory* (Immediate Recall)<br>*Verbal long-term memory* (Delayed Recall)	Education level<br>Etiology of HL<br>Duration of HL<br>HA use<br>Smoking status<br>Alcohol consumption<br>BMI<br>Arterial pressure	CI users showed meaningful gains in both immediate ($\beta = .09$, $p < .01$) and delayed recall ($\beta = .11$, $p < .001$). The quadratic slopes were negative ($\beta = -.09$, $p < .05$), suggesting an initial cognitive boost post-CI that leveled off after 2-y. In contrast, HRS and ELSA participants showed a linear cognitive decline over time and a more pronounced convex shape of decline for those with HL. Cognitive improvement in CI users was influenced by education and sex, but not by smoking, alcohol, or BMI.	CI supports memory improvement over the first 1–2-y, suggesting a booster effect in cognitive trajectories. However, this improvement seems to level off over time. CI may help counter early memory decline associated with hearing loss, but additional factors are needed for long-term dementia prevention.
**Young et al., 2023 [39]**<br>Prospective cohort study<br><br>2	> 65 years<br>Bilateral sensorineural HL<br>CI candidacy based on insurance approval	CI candidates<br>n = 55<br>77 [65-93]	- Baseline (pre-CI)<br>- 1-y post-CI<br>- 2-y post-CI	WRS in quiet (CNCw)<br>SRS in quiet (AzBio)<br>.	*Language, memory, executive function, calculations, abstractions, and visuospatial skills* (SAGE)	Education level<br>HA use	Limited change were reported in SAGE scores. However, patients within the group "SAGE passing" (score ≥17) had significantly better speech scores than patients within the SAGE fail group (score ≤16) at 1 and 2-y: 1-y CNC: 62.3% vs 51.0% ($p = .0423$); 1-y AzBio: 73.7% vs 61.2% ($p = .0345$); 2-y AzBio: 77.7% vs 52.3% ($p = .0033$).	Cognitive function measured by SAGE is a stronger predictor of post-CI speech recognition outcomes compared with age. While CI led to significant improvements in speech perception, it did not significantly improve cognition over 2 years. However, stable SAGE scores in the cognitively impaired group may indicate stabilization of cognitive decline.
**Mosnier et al., 2024 [74]**	≥ 60 years<br>Bilateral severe-to-profound HL	CI candidates<br>n = 98<br>(71.7 ± 7.6, 60-91)	- Baseline (pre-CI)<br>- 12-m post-CI<br>- 18-m post-CI	No speech perception measures due	*Global Cognitive screening* (MMSE)<br>*Processing speed* (Digit Symbol Coding)	Education Level<br>HRQoL (SSQ)	MMSE, TMT-B, or TUG scores post-CI had no meaningful changes. Meanwhile, Digit Symbol Coding improved significantly in the 60–64 age	CI significantly improved subjective HRQoL, attention and processing speed in younger older adults (60–

Prospective observational repeated-measures, single subject study<br><br>2	Postlingual HL<br>Unilateral CI candidacy<br>Normal cochlear anatomy<br>Complete electrode array insertion<br>No comorbidities limiting participation			to the multilingual nature of the study	*Attention, concentration, processing speed and cognitive flexibility* (TMT B)<br>*Executive function, balance* (TUG)	Depression (GDS-15)	group from baseline to 12-m and these gains remain at18-m. Cognitive flexibility and processing speed continued below normative data for 60–74 age groups, even after CI.	64), likely due to reallocation of cognitive resources. However, no significant change was observed in global cognition or executive function.
**Sarant et al., 2024 [64]**<br><br>Prospective longitudinal observational study<br><br>2	≥ 60 years severe-to-profound HL<br>Postlingual HL<br>CI candidacy<br>Normal cognition (MMSE ≥ 24 points)<br>No health conditions preventing follow-up	CI candidates<br>n = 101<br>73 [61–90]<br><br>AIBL Group<br>n = 100<br>74 [67–85]	CI candidates<br>- Baseline (pre-CI)<br>- 18-m post-CI<br>- 36-m post-CI<br>- 54-m post-CI<br><br>AIBL Group<br>- Baseline	PTA<br>WRS in quiet (CVCw)<br>SRS in noise (BKB)	*Global Cognitive screening* (MMSE)<br>Cogstate Brief Battery:<br>*Working Memory* (One Back Test)<br>*Attention* (Identification Test)<br>*Psychomotor function* (Detection Test)<br>*Visual learning* (One Card Learning)	Education level<br>Living status<br>HA use<br>QoL (HUI-3)<br>Anxiety and depression (HADS)<br>Loneliness (LSNS)<br>Medical Conditions<br>Apolipoprotein E (APOE) ε4 allele	CI group executive functions and working memory capacity achieve better performance at 54-m. Stable results were observed in attention, psychomotor function, and visual learning. Compared to the AIBL group, CI users had better cognitive trajectories in psychomotor function and visual attention.	CI may improve or stabilize cognitive performance, particularly executive function and memory, in older adults with severe hearing loss. Compared to untreated patients, CI users showed less cognitive decline, supporting CIs as a tool to delay age-related cognitive decline.
**Schauwecker et al., 2024 [75]**<br><br>Prospective study<br><br>2	≥ 55 years severe-to-profound HL<br>Postlingual HL<br>CI candidacy<br>High school education<br>No comorbidities limiting participation	CI candidates<br>n = 15<br>66.7 [53-76]	Neurocognitive assessment:<br>-Baseline (pre-CI)<br><br>Audiological assessment:<br>- 1-m post-CI<br>- 3-m post-CI	PTA<br>WRS in quiet (CNCw)<br>SRS in quiet and in noise (AzBio)	*Global Cognitive screening* (MMSE)<br>*Working Memory* (Digit span)<br>*Lexical and phonological processing* (TOWRE-2)<br>*Inhibitory control* (SCWT)	Socioeconomic status<br>HA use<br>Etiology of HL<br>Duration of HL	Working memory significantly predicted speech outcomes: AzBio in quiet (1-m; p ≤ .001, rho = .762,1), in noise (1-m; p ≤ .001, rho = .860) and in noise (3-m; *p* ≤ .001, rho = .786).<br>Digit Span correlated with CNC at 1- and 3-m but not after Bonferroni correction.<br>MMSE had moderate correlations with 1-month outcomes (CNC: *ρ* = .566, rho = .035), but none were significant after Bonferroni correction.<br>SCWT and TOWRE-2 did not correlate with any speech performance score.	Pre-CI working memory capacity is a strong predictor of early post-CI sentence recognition, especially in noise
**Vandenbroeke et al., 2024 [81]**<br><br>Prospective, longitudinal study<br><br>2	≥ 55 years old<br>Bilateral severe-to-profound HL<br>Postlingual HL<br>Unilateral CI candidacy<br>CI use ≥ 10 hours/day<br>No visual or medical conditions	CI candidates<br>n = 25<br>(69.8±8.6)	- Baseline (pre-CI)<br>- 1-y post-CI<br>- 2-y post-CI<br>- 3-y post-CI<br>- 4-y post-CI	PTA (Fletcher Index)<br>WRS in quiet (NVA list)<br>SRS in noise (LIST)	*Immediate Memory, Visuospatial Constructional, Language, Attention & Delayed Memory* (RBANS-H A&B).	Education level<br>Working status<br>HA use<br>Etiology of HL<br>Duration of HL<br>HRQoL (NCIQ, HISQUI, SSQ12)<br>Anxiety and Depression (HADS)<br>Personality (DS14)	Substantial cognitive gain at 1-year in RBANS-H total score: 92.78 → 98.35 (*p* < .001), Immediate Memory 94.13 → 105.39 (*p* = .005), Attention: 86.17 → 91.57 (*p* = .048), Delayed Memory 97.91 → 103.83 (*p* = .017).<br>At 4-y Immediate Memory still improved 94.13 → 101.91 (*p* = .02), while Visuospatial Memory declined 97.04 → 87.26 (*p* = .013).	CI improves cognition, especially memory and attention and QoL after - y, but cognitive gains are not sustained at 4-y, except in Immediate Memory. No cognitive decline was observed long-term, suggesting a stabilizing effect.
**Yoshida et al., 2025 [76]**<br>Retrospective cohort study<br><br>2	CI candidacy	CI candidates<br>n = 30<br>(70.1 ± 6.1)	- Baseline (pre-CI)<br>- 3.7-y post-CI	WRS in quiet (Japan Audiological Society)	*Global Cognitive screening* (MMSE)<br>*Reading* (ReaCT Kyoto)<br>*Visuospatial reasoning, executive function, and constructional ability* (Kohs block design)<br>Nonverbal reasoning (RCPM)	HA use<br>Duration of HL	The majority of patients showed stable or improvement in scores of KOHS and RCPM after CI. Pre-CI KOHS and RCPM demonstrated a significant positive correlation with post-CI SDS.	Cognitive tests can offer valuable information for predicting outcomes of CI. Moreover, cognitive function tends to remain stable over a long period after CI.

ADS-L: General Depression Scale–Long; AIBL: Australian Imaging, Biomarker and Lifestyle Flagship Study of Ageing; ALA cog: Alzheimer's Association Cognitive Test; AST: Attention switching task; BKB: Bamford–Kowal–Bench-like sentence list; BNT: Boston Naming Test; BVMT: Brief Visuospatial Memory Test- Revised; CANTAB: Cambridge Neuropsychological Test Automated Battery; CDT: Clock Drawing Test Clock; CERAD: Consortium to Establish a Registry for Alzheimer's Disease; CI: Cochlear Implant; CIRS-G: Cumulative Illness Rating Scale-Geriatri; CNCw: Consonant–Nucleus–Consonant word; CNS: Central Nervous System; CODEX: Cognitive Disorders Examination; CPR: Word Lists Learning and Recall; CRIq: Cognitive Reserve Index Questionnaire; CUNY: City University of New York; CVCw: Consonant–vowel–consonant words; d2: Test of Attention; DASS-21:Depression Anxiety Stress Scale; DMS: Delayed matching to sample; DS14: Type D personality questionnaire; FMT: Freiburg Monosyllabic Speech Test; GBI: Glasgow Benefit Inventory; GDS: Geriatric Depression Scale; GHSI: Glasgow Health Status Inventory; GMLT: Groton Maze Learning Test; IADL: Instrumental activities of daily living; HA: Hearing aids; HADS: Hospital Anxiety and Depression Scale; Hayling: Hayling Sentence Completion Test; HINT: Hearing in noise test; HISQUI-19: Hearing Implant Sound Quality Index-19;HRQoL: Hearing-Related Quality of Life; HSM: Hochmair-Desoyer, Schulz, Moser sentence test; HVLT-R: Hopkins Verbal Learning Test- Revised; iFlanker: incompatible Flanker; cFlanker: compatible Flanker; IPAQ: International Physical Activity Questionnaire; MBAA: Marginal Benefit for Acoustic Amplification; MMSE: Mini mental state examination; MoCA: Montreal Cognitive Assessment; MFTC: Multiple Features Target Cancellation; MOT: Motor screening task; MRI: Magnetic Resonance Imaging; MWI:

Mehrfachwahl-Wortschatz-Intelligenztest: NART-R: National adult reading test revised; NCIQ: Nijmegen Cochlear Implant Questionnaire; NH: Normal hearing; NVA: Nijmegen Verbal Learning Test-Auditory; LIST: Leuven Intelligibility Sentences Test; LSNS: Lubben Social Network Scale; OCLT: One Card Learning Task; OI: Oldenburg Inventory; OLSA: Oldenburg Sentence Test; PAL: Paired associates learning; PSQ: Perceived Stress Questionnaire; PTA: pure tone audiometry; QoL: Quality of Life; RCPM: Raven's colored progressive matrices; RAVLT: Rey Auditory Verbal Learning Task; RBANS: Repeatable Battery for the Assessment of Neuropsychological Functioning ; RBANS-H Repeatable Battery for the Assessment of Neuropsychological Status for Hearing impaired individuals; ReaCT Kyoto: Reading cognitive test Kyoto; ROCF: Rey Osterreith Complex Figure; SAGE: Self-Administered Gerocognitive Exam; SD: Standard deviation; SDS:Self-Rating Depression Scale; SDT: Speech Detection Threshold; SCWT: Stroop Color Word Test; SNHL: Sensorineural Hearing Loss; SNR: Signal to Noise Ratio; SSQ12: Speech, Spatial, and Qualities of hearing Scale–12; SRS: Sentence Recognition Score; SRT: Speech Recognition Threshold; SWM: Spatial working memory; TMT: Trail Making Test; TOPF: Test of Premorbid Functioning; TOWRE-2: Test of Word Reading Efficiency, version 2; TUG: Timed Up and Go; VRM: Verbal recognition memory; WASI: Wechsler Abbreviated Scale of Intelligence; WAIS-IV: Wechsler Adult Intelligence Scale 4th Edition; WHOQOL: World Health Organization Quality of Life Assessment for elderly people; WLLR: Word Lists Learning and Recall; WM: working memory; WRAT: Wide Range Achievement Test; WRS: Word Recognition Score.

References

28. Mosnier, I.; Bebear, J.-P.; Marx, M.; Fraysse, B.; Truy, E.; Lina-Granade, G.; Mondain, M.; Sterkers-Artières, F.; Bordure, P.; Robier, A.; et al. Improvement of Cognitive Function after Cochlear Implantation in Elderly Patients. *JAMA Otolaryngol Head Neck Surg* **2015**, *141*, 442–450, doi:10.1001/jamaoto.2015.129.

39. Young, A.; Fechtner, L.; Kim, C.; Nayak, N.; Kellermeyer, B.; Ortega, C.; Rende, S.; Rosenberg, S.; Wazen, J. Long-Term Cognition and Speech Recognition Outcomes after Cochlear Implantation in the Elderly. *Am J Otolaryngol* **2023**, *45*, 104071, doi:10.1016/j.amjoto.2023.104071.

50. Peterson, J.; Welch, V.; Losos, M.; Tugwell, P.; others The Newcastle-Ottawa Scale (NOS) for Assessing the Quality of Nonrandomised Studies in Meta-Analyses. *Ottawa: Ottawa Hospital Research Institute* **2011**, *2*, 1–12.

53. Cosetti, M.K.; Pinkston, J.B.; Flores, J.M.; Friedmann, D.R.; Jones, C.B.; Roland, J.T.; Waltzman, S.B. Neurocognitive Testing and Cochlear Implantation: Insights into Performance in Older Adults. *Clin Interv Aging* **2016**, *11*, 603–613, doi:10.2147/CIA.S100255.

54. Baranger, M.; Manera, V.; Sérignac, C.; Derreumaux, A.; Cancian, E.; Vandersteen, C.; Gros, A.; Guevara, N. Evaluation of the Cognitive Function of Adults with Severe Hearing Loss Pre- and Post-Cochlear Implantation Using Verbal Fluency Testing. *J Clin Med* **2023**, *12*, 3792, doi:10.3390/jcm12113792.

55. Gurgel, R.K.; Duff, K.; Foster, N.L.; Urano, K.A.; deTorres, A. Evaluating the Impact of Cochlear Implantation on Cognitive Function in Older Adults. *The Laryngoscope* **2022**, *132*, S1–S15, doi:10.1002/lary.29933.

56. Völter, C.; Götze, L.; Dazert, S.; Falkenstein, M.; Thomas, J.P. Can Cochlear Implantation Improve Neurocognition in the Aging Population? *Clin Interv Aging* **2018**, *13*, 701–712, doi:10.2147/CIA.S160517.

57. Völter, C.; Götze, L.; Haubitz, I.; Müther, J.; Dazert, S.; Thomas, J.P. Impact of Cochlear Implantation on Neurocognitive Subdomains in Adult Cochlear Implant Recipients. *Audiol Neurootol* **2021**, *26*, 236–245, doi:10.1159/000510855.

58. Völter, C.; Götze, L.; Bajewski, M.; Dazert, S.; Thomas, J.P. Cognition and Cognitive Reserve in Cochlear Implant Recipients. *Front Aging Neurosci* **2022**, *14*, 838214, doi:10.3389/fnagi.2022.838214.

59. Völter, C.; Goetze, L.; Dazert, S.; Thomas, J.P.; Kamin, S.T. Longitudinal Trajectories of Memory among Middle-Aged and Older People with Hearing Loss: The Influence of Cochlear Implant Use on Cognitive Functioning. *FRONTIERS IN AGING NEUROSCIENCE* 2023, *15*.

60. Jayakody, D.M.P.; Peter L. Friedland; Nel, E.; Martins, R.N.; Atlas, M.D.; Sohrabi, H.R. Impact of Cochlear Implantation on Cognitive Functions of Older Adults: Pilot Test Results. *Otol Neurotol* **2017**, *38*, e289–e295, doi:10.1097/MAO.0000000000001502.

61. Huber, M.; Roesch, S.; Pletzer, B.; Lukaschyk, J.; Lesinski-Schiedat, A.; Illg, A. Can Cochlear Implantation in Older Adults Reverse Cognitive Decline Due to Hearing Loss? *Ear Hear* **2021**, *42*, 1560–1576, doi:10.1097/AUD.0000000000001049.

62. Ambert-Dahan, E.; Routier, S.; Marot, L.; Bouccara, D.; Sterkers, O.; Ferrary, E.; Mosnier, I. Cognitive Evaluation of Cochlear Implanted Adults Using CODEX and MoCA Screening Tests. *Otol Neurotol* **2017**, *38*, e282–e284, doi:10.1097/MAO.0000000000001464.

63. Sarant, J.; Harris, D.; Busby, P.; Maruff, P.; Schembri, A.; Dowell, R.; Briggs, R. The Effect of Cochlear Implants on Cognitive Function in Older Adults: Initial Baseline and 18-Month Follow Up Results for a Prospective International Longitudinal Study. *Front. Neurosci.* **2019**, *13*, doi:10.3389/fnins.2019.00789.

64. Sarant, J.Z.; Busby, P.A.; Schembri, A.J.; Briggs, R.J.S.; Masters, C.L.; Harris, D.C. COCHLEA: Longitudinal Cognitive Performance of Older Adults with Hearing Loss and Cochlear Implants at 4.5-Year Follow-Up. *Brain Sciences* **2024**, *14*, 1279, doi:10.3390/brainsci14121279.

65. Castiglione, A.; Benatti, A.; Velardita, C.; Favaro, D.; Padoan, E.; Severi, D.; Pagliaro, M.; Bovo, R.; Vallesi, A.; Gabelli, C.; et al. Aging, Cognitive Decline and Hearing Loss: Effects of Auditory Rehabilitation and Training with Hearing Aids and Cochlear Implants on Cognitive Function and Depression among Older Adults. *Audiol Neurootol* **2016**, *21 Suppl 1*, 21–28, doi:10.1159/000448350.

66. Vasil, K.J.; Ray, C.; Lewis, J.; Stefancin, E.; Tamati, T.N.; Moberly, A.C. How Does Cochlear Implantation Lead to Improvements on a Cognitive Screening Measure? *J Speech Lang Hear Res* **2021**, *64*, 1053–1061, doi:10.1044/2020_JSLHR-20-00195.

67. Sonnet, M.-H.; Montaut-Verient, B.; Niemier, J.-Y.; Hoen, M.; Ribeyre, L.; Parietti-Winkler, C. Cognitive Abilities and Quality of Life After Cochlear Implantation in the Elderly. *Otol Neurotol* **2017**, *38*, e296–e301, doi:10.1097/MAO.0000000000001503.

68. Mosnier, I.; Vanier, A.; Bonnard, D.; Lina-Granade, G.; Truy, E.; Bordure, P.; Godey, B.; Marx, M.; Lescanne, E.; Venail, F.; et al. Long-Term Cognitive Prognosis of Profoundly Deaf Older Adults After Hearing Rehabilitation Using Cochlear Implants. *J Am Geriatr Soc* **2018**, *66*, 1553–1561, doi:10.1111/jgs.15445.

69. Anzivino, R.; Conti, G.; Di Nardo, W.; Fetoni, A.R.; Picciotti, P.M.; Marra, C.; Guglielmi, V.; Fortunato, S.; Forli, F.; Paludetti, G.; et al. Prospective Evaluation of Cognitive Functions After Rehabilitation With Cochlear Implant or Hearing Aids: Preliminary Results of a Multicentric Study on Elderly Patients. *Am J Audiol* **2019**, *28*, 762–774, doi:10.1044/2019_AJA-HEAL18-18-0176.

70. Sorrentino, T.; Donati, G.; Nassif, N.; Pasini, S.; Redaelli de Zinis, L.O. Cognitive Function and Quality of Life in Older Adult Patients with Cochlear Implants. *Int. J. Audiol.* **2020**, *59*, 316–322, doi:10.1080/14992027.2019.1696993.

71. Herzog, J.A.; Buchman, C.A.; Kallogjeri, D.; Chen, S.; Wick, C.; Durakovic, N.; Shew, M.A. Cognitive Assessment in Elderly Cochlear Implant Recipients: Long-Term Analysis. *Laryngoscope* **2022**, doi:10.1002/lary.30466.

72. Ohta, Y.; Imai, T.; Maekawa, Y.; Morihana, T.; Osaki, Y.; Sato, T.; Okazaki, S.; Hanamoto, M.; Suwa, K.; Takeya, Y.; et al. The Effect of Cochlear Implants on Cognitive Function in Older Adults: A Prospective, Longitudinal 2-Year Follow-up Study. *Auris Nasus Larynx* **2022**, *49*, 360–367, doi:10.1016/j.anl.2021.09.006.

73. Zucca, M.; Albera, A.; Albera, R.; Montuschi, C.; Della Gatta, B.; Canale, A.; Rainero, I. Cochlear Implant Results in Older Adults with Post-Lingual Deafness: The Role of "Top-Down" Neurocognitive Mechanisms. *Int J Environ Res Public Health* **2022**, *19*, 1343, doi:10.3390/ijerph19031343.

74. Mosnier, I.; Belmin, J.; Cuda, D.; Manrique Huarte, R.; Marx, M.; Ramos Macias, A.; Khnifes, R.; Hilly, O.; Bovo, R.; James, C.J.; et al. Cognitive Processing Speed Improvement after Cochlear Implantation. *Front. Aging Neurosci.* **2024**, *16*, doi:10.3389/fnagi.2024.1444330.

75. Schauwecker, N.; Tamati, T.N.; Moberly, A.C. Predicting Early Cochlear Implant Performance: Can Cognitive Testing Help? *Otology & Neurotology Open* **2024**, *4*, e050, doi:10.1097/ONO.0000000000000050.

76. Yoshida, T.; Kobayashi, M.; Hara, D.; Taniguchi, R.; Fukunaga, Y.; Sone, M. Cognitive Function and Speech Outcomes after Cochlear Implantation in Older Adults. *Front. Neurol.* **2025**, *16*, doi:10.3389/fneur.2025.1630946.

77. Claes, A.J.; Van de Heyning, P.; Gilles, A.; Van Rompaey, V.; Mertens, G. Cognitive Performance of Severely Hearing-Impaired Older Adults Before and After Cochlear Implantation: Preliminary Results of a Prospective, Longitudinal Cohort Study Using the RBANS-H. *Otol Neurotol* **2018**, *39*, e765–e773, doi:10.1097/MAO.0000000000001936.

78. Mertens, G.; Andries, E.; Claes, A.J.; Topsakal, V.; Van de Heyning, P.; Van Rompaey, V.; Calvino, M.; Cuadrado, I.S.; Muñoz, E.; Gavilán, J.; et al. Cognitive Improvement after Cochlear Implantation in Older Adults with Severe or Profound Hearing Impairment: A Prospective, Longitudinal, Controlled, Multicenter Study. *Ear and hearing* **2021**, *42*, 606.

79. Calvino, M.; Sánchez-Cuadrado, I.; Gavilán, J.; Lassaletta, L. The Effect of Risk Factors on Cognition in Adult Cochlear Implant Candidates with Severe to Profound Hearing Loss. *Frontiers in Psychology* **2022**, *13*, doi:10.3389/fpsyg.2022.837366.

80. Andries, E.; Bosmans, J.; Engelborghs, S.; Cras, P.; Vanderveken, O.M.; Lammers, M.J.W.; van de Heyning, P.H.; Van Rompaey, V.; Mertens, G. Evaluation of Cognitive Functioning Before and After Cochlear Implantation in Adults Aged 55 Years and Older at Risk for Mild Cognitive Impairment. *JAMA OTOLARYNGOLOGY-HEAD & NECK SURGERY* 2023, *149*, 310–316.

81. Vandenbroeke, T.; Andries, E.; Lammers, M.J.; Van de Heyning, P.; Hofkens-Van den Brandt, A.; Vanderveken, O.; Van Rompaey, V.; Mertens, G. Cognitive Changes Up to 4 Years After Cochlear Implantation in Older Adults: A Prospective Longitudinal Study Using the RBANS-H. *Ear and hearing* **2024**, doi:10.1097/AUD.0000000000001583.

82. Knopke, S.; Schubert, A.; Häussler, S.M.; Gräbel, S.; Szczepek, A.J.; Olze, H. Improvement of Working Memory and Processing Speed in Patients over 70 with Bilateral Hearing Impairment Following Unilateral Cochlear Implantation. *J Clin Med* **2021**, *10*, 3421, doi:10.3390/jcm10153421.

83. Haeussler, S.M.; Stankow, E.; Knopke, S.; Szczepek, A.J.; Olze, H. Sustained Cognitive Improvement in Patients over 65 Two Years after Cochlear Implantation. *BRAIN SCIENCES* 2023, *13*.

84. Zhan, K.Y.; Lewis, J.H.; Vasil, K.J.; Tamati, T.N.; Harris, M.S.; Pisoni, D.B.; Kronenberger, W.G.; Ray, C.; Moberly, A.C. Cognitive Functions in Adults Receiving Cochlear Implants: Predictors of Speech Recognition and Changes After Implantation. *Otol Neurotol* **2020**, *41*, e322–e329, doi:10.1097/MAO.0000000000002544.
