# Supplementary material for: Neuropsychological Assessments to Explore the Cognitive Impact of Cochlear Implants: A Scoping Review
Source: J Clin Med. 2025 Oct 27;14(21):7628. doi: 10.3390/jcm14217628 (PMC12608580; doi:10.3390/jcm14217628)
Supplement: Supplementary file 1 [file jcm-14-07628-s001.zip › Table S2. Characteristics and Main Findings of Cross-sectional Studies Assessing Neuropsychological and Audiological Outcomes in Cochlear Implant Users and Control Groups.pdf]

**Table S2. Characteristics and Main Findings of Cross-sectional Studies Assessing Neuropsychological and Audiological Outcomes in Cochlear Implant Users and Control Groups.**

| **Author(s), year**<br><br>**Design and Level of Evidence** | Inclusion / Exclusion criteria | Participants *Age (mean ± SD and/or range)* | Time of Testing | Audiological assessment | Cognitive test (*domain measured*) | Other Variables and tests | Cognitive findings | Principal conclusion |
|---|---|---|---|---|---|---|---|---|
| **Castiglione et al., 2016 [56]**<br><br>Cross-sectional studies<br><br>2 | ≥ 65 years<br>moderate-to-severe HL<br>Group C: > (1 - 2)-y unilateral HA users<br>Group D: No treatment<br>Group F:NH controls | Participants<br>n= 125 (65-89 years)<br><br>Group C: 15<br>Group D: 15<br>Group F: 20 | Cross-sectional for groups C, D and F | PTA<br>SDT and SRT<br>with disyllabic/trisyllabic words and SRS<br>(Nombre) | *Global cognition* (MoCA) | Age at implantation<br>Depression<br>(GDS) | Groups C and F showed significantly better outcomes in cognitive function and depression compared to group D, which exhibited impaired cognition and depressive symptoms. | Cognitive and memory outcomes in patients with HA and NH, were better than in mild-to-moderate HL subjects without hearing rehabilitation. |
| **Hua et al., 2017 [98]**<br><br>Cross-sectional observational study<br><br>3 | >18 years<br>postlingual progressive HL<br>bimodal stimulation (CI + HA) ≥8 hours<br>≥1 year CI experience<br>No severe medical disorder | Bimodal users<br>n = 17<br>(53.5 ± 14, [28-74]) | Single time-point<br>(≥1 year CI experience) | WRS in quiet<br>(SPB word list)<br>SRS in noise (HINT) | *Processing speed, attention, executive control, and task-switching ability* (TMT A&B)<br>*Working memory capacity* (Reading Span Test) | Duration of HL<br>HA use<br>CI use | TMT B scores correlated with speech recognition across all listening conditions: CI-only WRS in quiet ( $r = -.52$, $p < .05$), bimodal WRS in quiet ($r = -.75$, $p < .01$) and HINT $r = .55$, $p < .05$) . Reading span test significantly correlated with bimodal WRS in quiet ($r = .71$, $p < .01$) but not with HINT. | Executive function capacities (TMT-B, RST) are associated with speech intelligibility in quiet and noise, especially in bimodal users. |
| **Claes et al., 2018 [92]**<br><br>Cross-sectional study<br><br>2 | CI users inclusion:<br>≥ 50 years<br>Postlingual severe-to-profound HL<br>Unilateral CI<br>≥1 year of CI experience<br>No severe medical disorder<br><br>NH inclusion:<br>≥ 50 years<br>No severe medical disorder | CI users<br>n = 61<br>71.0 [58.3 – 93.9]<br><br>NH controls<br>n = 81<br>69.9 [50.1 - 87.1] | Single time-point<br>(>12 months CI experience) | WRS in quiet<br>(NVA lists or Dutch Society of Audiology)<br>SRS in noise (LIST)<br>SRT (NVA list) in quiet and in noise. | *Immediate Memory, Visuospatial Constructional, Language, Attention & Delayed Memory* (RBANS-H) | Age<br>Sex<br>Education<br>HA use<br>CI use | CI recipients performed lower in RBANS-H scores than NH controls with correction of age, sex, and education. Worse cognition scores were associated with decreased speech perception in quiet and in noise, independently of age. Lower SRT was associated with higher cognitive performance in both groups. | CI recipients with at least one year of experience exhibit below normal cognitive functioning, independent of the effects of age, sex, and education. |
| **Kramer et. al., 2018 [94]**<br><br>Cross-sectional study<br><br>2b | Severe-to-profound HL<br>Postlingual HL<br>CI candidacy or ≥1 year of CI<br>Normal Cognition (MMSE ≥ 26)<br>Word reading (WRAT ≥75)<br>NH control matched on age | Experienced CI users (ECI)<br>n = 43<br>(67.7 ± 9.3)<br><br>CI candidacy (CIC)<br>n = 19<br>(69.8± 9.8)<br><br>NH controls<br>n = 40<br>(66.8 ±6.6) | Single time-point<br>(>18 months of CI experience & pre-CI) | PTA | *Working memory* (Visual digit span, visual object span, and visual symbol span)<br>*Information-Processing speed and inhibitory control* (SCWT)<br>*Nonverbal reasoning* (Raven's)<br>*Speed of phonological and lexical access* (TOWRE-2)<br>*Verbal learning and memory* (CVLT-II) | Socioeconomic status<br>Reading and vocabulary knowledge (WordFAM)<br>HL etiology<br>Comorbidities | NH patients has greater scores than ECI users and CICs in non-auditory cognitive tasks, including nonverbal reasoning, processing speed, lexical access, and verbal memory. Adjusting for SES and reading proficiency (WRAT) reduced some cognitive differences between groups, but significant differences remained in working memory (symbol span), nonverbal reasoning (Raven's), speed of lexical access (TOWRE-2), and verbal learning and memory (CVLT-II total recall). Meaningful differences were not consistently found between CICs and ECIs. | Although NH participants generally performed better than ECI and CIC subjects, no large consistent discrepancies were reported in cognitive functions between individuals with ECI and CIC individuals were found. |
| **Moberly et al., 2018 [30]**<br><br>Cross-sectional study<br><br>2 | Postlingual HL<br>CI candidacy<br>No retrocochlear disorders or anatomical limitations<br>Normal cognition<br>No severe medical disorder | CI candidacy<br>n = 31<br>(69.6 ± 10.9, [49-94]) | Single time-point<br>(pre-CI) | PTA (best-aided, residual hearing)<br>SRS in quiet and babble noise<br>(AzBio)<br>SRS in quiet and babble noise (CUNY) | *Global* Cognitive screening (MMSE)<br>*Verbal working memory (*Digit Span)<br>*Inhibition and processing speed* (SCWT)<br>*Nonverbal reasoning* (Raven's) | Education level<br>Socioeconomic status<br>Duration of HL<br>HA use<br>Etiology of HL | Working memory (Digit Span) significantly predicted SRS in babble measured though AzBio, independent of PTA. Similarly, nonverbal reasoning ( Raven's) predicted SRS in quiet as measured through CUNY. | Post-CI SRS in quiet and in noise are associated by neurocognitive abilities like nonverbal reasoning and working memory, which should be considered when assessing CI candidacy. |

| | | | | | | | | |
|---|---|---|---|---|---|---|---|---|
| | No visual impairment | | | | *Lexical/phonological access and processing speed* (TOWRE)<br>*Reading Skills (*WRAT) | | | |
| **Giallini et al., 2023 [93]**<br><br>Observational study<br><br>3 | ≥ 60 years<br>Postlingual severe-to-profound HL<br>≥1-y of CI experience<br>No severe medical disorder | CI users<br>n = 30 CI<br>(73.4 ± 6.6) | Single time-point<br>( >12 months of CI experience) | PTA<br>WRS in quiet (Turrini)<br>SRS in quiet and noise (Burdo/Orsi)<br>ABR<br>OAE | *Global Cognitive screening* (MoCA)<br>*Intellectual disability* (CPMs)<br>*Short-term memory and simple working memory* (Digit Span)<br>*Verbal working memory* (CWMT)<br>*Attention (*RBANS attention subsection) | Education level<br>Age<br>Duration of HL<br>Etiology of HL<br>HA use<br>CI experience<br>Anxiety (STAI-Y) | High attention performers had markedly better performance in all working memory tasks, attention significantly correlated with CWMT (ρ = .67), Digit Span (Forward ρ = .74, Backwards: ρ = .53). Attention also positively correlated with educational level (ρ = .60), predicted SRS in noise (SNR+10: R = 0.53, $p$ = .017). Working memory showed trend-level significance for predicting SRS in noise (SNR+5: R = 0.47, $p$ = .019)- | Attention and working memory are significantly associated with better speech perception in noise among elderly CI users. High attentional capacity may reduce cognitive load during listening and improve auditory-verbal processing. |
| **Huber et al., 2023 [95]**<br><br>Cohort study<br><br>3 | 25–75 years<br>Bilateral severe-to-profound HL<br>CI candidacy<br>No significant visual impairment<br>No anticholinergic medication | Younger CI candidates<br>n= 17<br>(45.76 ± 9.47, 25–54)<br><br>Older CI candidates<br>n= 44<br>(65.18 ± 6.24, 55–75) | Single time-point<br>(pre-CI) | PTA<br>WRS in quiet (FMT) | *Visual episodic memory* (NVLT)<br>*Attention and working* memory (N-Back Test)<br>*Inhibitory control* (Go/No-Go Test)<br>*Cognitive flexibility* (TMT A & B)<br>*Phonemic and semantic verbal fluency* (RWT) | Education level<br>HA use<br>Duration of HL<br>Etiology of HL<br>HRQoL (APHAB)<br>Depression (BDI-II)<br>Comorbidities | No significant correlations were identified between depressive symptoms, PTA, and cognitive performance. However, subjective hearing ability in quiet as reported in APHAB correlated positively with visual episodic memory, phonemic fluency, and cognitive flexibility. Aversive sounds (APHAB) were significantly associated with semantic fluency and inhibition. | No mediation effect of depression was found between HL and cognitive performance. However, self-reported communication abilities in quiet situations were significantly associated with cognitive abilities. |
| **Schvartz-Leyzac et al., 2023 [91]**<br><br>Prognostic study<br><br>3 | > 18 years<br>≥ 6-m of CI experience<br>Perilingually or postlingually HL<br>No HA use<br>No vision impairment | CI users<br>n = 20<br>66 [28-80] | Single time-point<br>(> 6 months of CI experience) | SRS in noise<br>(CUNY) | NIH Toolbox Cognition Battery<br>*Attention and executive functioning* (Flanker)<br>*Episodic memory* (PSMT)<br>*Working memory* (LSWM)<br>*Language* (PVT,ORRT)<br>*Executive functioning* (DCCS)<br>*Processing speed* (PCPS) | Education Level<br>Socioeconomic status<br>HA use<br>CI use | A mild association between SRS in noise and the Pattern Comparison Processing Speed Test ($R^2$ = 0.28, β = –0.23, $p$ = .01) was found.<br>No significant associations for working memory, episodic memory, or executive function with speech performance. | Older CI users show worse SRS, which is significantly associated with slower processing speed. The adapted NIH Toolbox (visual version) is feasible for assessing cognition in CI users and may help identify factors influencing complex speech understanding. |
| **Ceuleers et al., 2024 [96]**<br><br>Observational study<br><br>3 | Moderate-to-severe or severe-to-profound HL<br>≥ 1-y HA and CI users<br>MoCA ≥ 23 for participants aged ≥60<br>No vision impairment<br>Normal Hearing | CI users<br>n = 31<br>(58.86 ± 14.28)<br><br>HA users<br>n = 31<br>(59.31 ± 14.06)<br><br>NH control group<br>n = 31<br>(58.76 ± 14.49) | Single time-point<br>(>12 months HA and CI experience) | PTA<br>SRT<br>WRS in quiet (Pb70)<br>SRS in noise (BLU word lists)<br>Audio visual SRS (TAUVIS) | *Working memory and verbal processing speed* (LNST)<br>*Selective attention (LDT)*<br>*Cognitive flexibility and inhibition* (Auditory SWCT) | Education level<br>HA use<br>CI use<br>Etiology of HL<br>Duration of HL | NH group had markedly higher scores in working memory, processing speed, attention, and cognitive flexibility vs. CI users (all $p$ < .01). HA users scored better than CI users for Stroop-words ($p$ = .013). While no substantial differences was found in Stroop interference score across groups. | NH individuals outperformed both HA and CI users on cognitive and auditory tasks. CI users showed greater audiovisual gain but poorer cognitive scores independently of educational level. |
| **Moberly et al., 2025 [97]**<br><br>Cross-sectional study<br><br>3 | Moderate-to-severe HL<br>≥ 1-y CI users<br>Normal Cognition (MMSE ≥ 26)<br>Word reading (WRAT ≥75)<br>No vision impairment<br>Normal Hearing | CI users<br>n = 54<br>(66.6 ± 9.4, 45-87]<br>NH control group<br>n = 43<br>(66.8 ± 6.6, 50-81) | Single time-point<br>(>12 month of CI) | PTA<br>WRS in quiet (CID-W22)<br>SRS in quiet (IEEE)<br>SRS in quiet (PRESTO)<br>Audio visual SRS (CUNY)<br>Noise-Vocoding | *Vocabulary Size* (WordFAM)<br>*Working memory (*Visual Digit Span)<br>*Inhibition and processing speed* (SCWT)<br>*Nonverbal reasoning* (Raven's) | HA use<br>Duration of HL<br>Socioeconomic status | NH outperformed CI users in working memory capacity, speed of lexical access, nonverbal reasoning, and vocabulary size. Speed of lexical access is positively associated with WRS in quiet in CI users ($p$ = .01) and SRS in quiet (PRESTO). For the same group, speed of lexical access and nonverbal reasoning correlated with SRS in quiet (IEEE, both $p$ = .01). | The language characteristics and complexity of the speech materials used in testing influence how different neurocognitive abilities contribute to speech recognition. |

ABR: Auditory Brainstem Response; AST: Attention switching task; BDI: Beck's Depression Inventory; CI: Cochlear Implant; CID: Central Institute of the Deaf; CPMs: Colored Progressive Matrices Raven's; CUNY: City University of New York; CVLT- II: California Verbal Learning Test, version II; CWMT: Cogmed Working Memory Training; DCCST: Dimensional Change Card Sort Test; FMT: Freiburg Monosyllabic Speech Test; GDS: Geriatric Depression Scale; HA: Hearing aids; HINT: Hearing in noise test; HRQoL: Hearing-Related Quality of Life; IEEE: Institute of Electrical and Electronics Engineers; MMSE: Mini mental state examination; MoCA: Montreal Cognitive Assessment; NH: Normal hearing; NVA:Nederlandse Vereniging voor Audiologie; NVLT: Non-Verbal Learning Test; LDT: Letter Detection Test; LDST: Letter Digit Substitution test; LIST: Leuven Intelligibility Sentences Test; LNST: Letter-Number Sequencing Task; LSWM: List Sorting Working Memory Test; OAE: Otoacoustic Emissions; ORRT: Oral Reading Recognition Test; PCPS: Pattern Comparison Processing Speed; PSMT: Picture Sequence Memory Test; PTA: pure tone audiometry; PVT: Picture Vocabulary Test; QoL: Quality of Life; RBANS: Repeatable Battery for the Assessment of Neuropsychological Functioning ; RBANS-H Repeatable Battery for the Assessment of Neuropsychological Status for Hearing impaired individuals; RWT: Regensburg Word Test; SAGE: Self-Administered Gerocognitive Exam; SCWT: Stroop Color Word Test; SD: Standard deviation; SDT: Speech Detection Threshold; SES: Socioeconomic status; SNHL: Sensorineural Hearing Loss; SNR: Signal to Noice Ratio; SPB: Swedish phonemically balanced; SRS: Sentence Recognition Score; SRT: Speech Recognition Threshold; STAI-Y: State-Trait Anxiety Inventory for Adults; TAUVIS: Test for Audiovisual Speech Perception; TMT: Trail Making Test; TOWRE: Test of Word Reading Efficiency ; WRAT: Wide Range Achievement Test; WRS: Word Recognition Score.

## References


30. Moberly, A.C.; Castellanos, I.; Mattingly, J.K. Neurocognitive Factors Contributing to Cochlear Implant Candidacy. Otol Neurotol 2018, 39, e1010–e1018, doi:10.1097/MAO.0000000000002052.

65. Castiglione, A.; Benatti, A.; Velardita, C.; Favaro, D.; Padoan, E.; Severi, D.; Pagliaro, M.; Bovo, R.; Vallesi, A.; Gabelli, C.; et al. Aging, Cognitive Decline and Hearing Loss: Effects of Auditory Rehabilitation and Training with Hearing Aids and Cochlear Implants on Cognitive Function and Depression among Older Adults. Audiol Neurootol 2016, 21 Suppl 1, 21–28, doi:10.1159/000448350.

91. Schvartz-Leyzac, K.C.; Giordani, B.; Pfingst, B.E. Association of Aging and Cognition With Complex Speech Understanding in Cochlear-Implanted Adults Use of a Modified National Institutes of Health (NIH) Toolbox Cognitive Assessment. *JAMA OTOLARYNGOLOGY-HEAD & NECK SURGERY* 2023, *149*, 239–246.

92. Claes, A.J.; Van de Heyning, P.; Gilles, A.; Hofkens-Van den Brandt, A.; Van Rompaey, V.; Mertens, G. Impaired Cognitive Functioning in Cochlear Implant Recipients Over the Age of 55 Years: A Cross-Sectional Study Using the Repeatable Battery for the Assessment of Neuropsychological Status for Hearing-Impaired Individuals (RBANS-H). *Front Neurosci* **2018**, *12*, 580, doi:10.3389/fnins.2018.00580.

93. Giallini, I.; Inguscio, B.M.S.; Nicastri, M.; Portanova, G.; Ciofalo, A.; Pace, A.; Greco, A.; D'Alessandro, H.D.; Mancini, P. Neuropsychological Functions and Audiological Findings in Elderly Cochlear Implant Users: The Role of Attention in Postoperative Performance. *AUDIOLOGY RESEARCH* 2023, *13*, 236–253.

94. Kramer, S.; Vasil, K.J.; Adunka, O.F.; Pisoni, D.B.; Moberly, A.C. Cognitive Functions in Adult Cochlear Implant Users, Cochlear Implant Candidates, and Normal-Hearing Listeners. *Laryngoscope Investig Otolaryngol* **2018**, *3*, 304–310, doi:10.1002/lio2.172.

95. Huber, M.; Reuter, L.; Weitgasser, L.; Pletzer, B.; Rösch, S.; Illg, A. Hearing Loss, Depression, and Cognition in Younger and Older Adult CI Candidates. *Front. Neurol.* **2023**, *14*, doi:10.3389/fneur.2023.1272210.

96. Ceuleers, D.; Keppler, H.; Degeest, S.; Baudonck, N.; Swinnen, F.; Kestens, K.; Dhooge, I. Auditory, Visual, and Cognitive Abilities in Normal-Hearing Adults, Hearing Aid Users, and Cochlear Implant Users. *EAR AND HEARING* 2024, *45*, 679–694.

97. Moberly, A.C.; Du, L.; Tamati, T.N. Individual Differences in the Recognition of Spectrally Degraded Speech: Associations With Neurocognitive Functions in Adult Cochlear Implant Users and With Noise-Vocoded Simulations. *Trends Hear* **2025**, *29*, 23312165241312449, doi:10.1177/23312165241312449.

98. Hua, H.; Johansson, B.; Magnusson, L.; Lyxell, B.; Ellis, R.J. Speech Recognition and Cognitive Skills in Bimodal Cochlear Implant Users. *J Speech Lang Hear Res* **2017**, *60*, 2752–2763, doi:10.1044/2017_JSLHR-H-16-0276.
