# Supplementary material for: Neuropsychological Assessments to Explore the Cognitive Impact of Cochlear Implants: A Scoping Review
Source: J Clin Med. 2025 Oct 27;14(21):7628. doi: 10.3390/jcm14217628 (PMC12608580; doi:10.3390/jcm14217628)
Supplement: Supplementary file 1 [file jcm-14-07628-s001.zip › Table S3. Newcastle-Ottawa Scale (NOS).pdf]

Supplementary Table S3. Newcastle-Ottawa Scale (NOS) for assessment of Risk of Bias

	**Selection**				**Comparability**	**Outcome**			
**Author, year**	Representativeness of the exposed cohort	Selection of the non-exposed cohort	Ascertainment of exposure	Demonstration That Outcome of Interest Was Not Present at Start of Study	Comparability of Cohorts on the Basis of the Design or Analysis	Assessment	Follow-up long enough	Adequacy of Follow Up of Cohorts	**NOS Score**
Mosnier et al., 2015 [28]	*	-	*	*	**	*	*	*	8
Castiglione et al., 2016 [53]	*	*	*	*	*	*	*	*	8
Cosetti et al., 2016 [49]	-	-	*	*	**	*	*	*	7
Hua et al., 2017	*	-	*	*	*	*	*	*	7
Ambert-Dahan et al., 2017 [52]	*	-	*	*	**	*	*	*	8
Jayakody et al., 2017 [66]	*	-	*	*	*	*	*	*	7
Sonnet et al., 2017 [61]	*	-	*	*	**	*	*	*	8
Claes et al., 2018 [56]	*	-	*	*	**	*	*	*	8
Claes et al., 2018 [93]	*	*	*	*	**	*	*	*	9
Kramer et al., 2018 [90]	*	*	*	*	**	*	*	*	9
Moberly et al., 2018 [30]	*	-	*	*	**	*	*	*	8
Mosnier et al., 2018 [63]	*	-	*	*	**	*	*	*	8
Völter et al., 2018 [70]	*	-	*	*	*	*	*	*	7
Anzivino et al., 2019 [64]	*	-	*	*	**	*	*	*	8
Sarant et al., 2019 [80]	*	-	*	*	**	*	*	*	8
Sorrentino et al., 2020 [60]	*	*	*	*	**	*	*	*	9
Zhan et al., 2020 [78]	*	*	*	*	*	*	*	*	8
Huber et al., 2021[73]	*	*	*	*	**	*	*	*	9
Knopke et al., 2021 [76]	*	*	*	*	**	*	*	*	9

Mertens et al., 2021 [57]	*	-	*	*	**	*	*	*	8
Vasil et al., 2021 [54]	*	-	*	*	*	*	*	*	7
Völter et al., 2021 [67]	*	*	*	*	**	*	*	*	9
Calvino et al., 2022 [65]	*	-	*	*	*	*	*	*	7
Gurgel et al., 2022 [51]	*	-	*	*	**	*	*	*	8
Herzog et al., 2022 [62]	*	-	*	*	*	*	*	*	7
Ohta et al., 2022 [59]	*	-	*	*	*	*	*	*	7
Völter et al., 2022 [68]	*	-	*	*	**	*	*	*	8
Zucca et al., 2022 [82]	*	-	*	*	*	*	*	*	7
Andries et al., 2023 [55]	*	-	*	*	**	*	*	*	8
Baranger et al., 2023 [50]	*	-	*	*	**	*	-	-	6
Giallini et al., 2023 [89]	*	-	*	*	**	*	*	*	8
Haeussler et al., 2023 [77]	*	-	*	*	*	*	*	*	8
Huber et al., 2023 [101]	*	-	*	*	**	*	*	*	8
Schvartz-Leyzac et al., 2023 [91]	*	-	*	*	*	*	*	*	7
Völter et al., 2023 [69]	*	*	*	*	**	*	*	*	9
Young et al., 2023 [39]	*	-	*	*	*	*	*	*	7
Ceuleers et al., 2024 [88]	*	*	*	*	*	*	*	*	8
Mosnier et al., 2024 [83]	*	-	*	*	**	*	*	*	8
Sarant et al., 2024 [71]	*	*	*	*	**	*	*	*	9
Schauwecker et al., 2024 [84]	*	-	*	*	**	*	*	*	8
Vandenbroeke et al., 2024 [58]	*	-	*	*	**	*	*	*	8

Moberly et al. 2025 [92]	*	*	*	*	*	*	*	*	8
Yoshida et al., 2025 [85]	*	-	*	*	*	*	*	*	7

Explanation: The maximum score is 9 stars. Each criterion earns one star, except for comparability, which can receive up to two stars.

7-9 stars: Low Risk

4-6 stars: Moderate Risk

0-3 stars: High Risk
