# Supplementary material for: Neuropsychological Assessments to Explore the Cognitive Impact of Cochlear Implants: A Scoping Review
Source: J Clin Med. 2025 Oct 27;14(21):7628. doi: 10.3390/jcm14217628 (PMC12608580; doi:10.3390/jcm14217628)
Supplement: Supplementary file 1 [file jcm-14-07628-s001.zip › Table S4. Description of the Cognitive Test Used in the Scoping Review.pdf]

Supplementary Table S4. Description of the Cognitive Tests Used in the Scoping Review.

Test	Cognitive Domains Assessed	Objective	Score descriptions and interpretation	Reference
AlaCog	Global cognition, attention, memory, executive function, language, visuospatial abilities	Computer-based cognitive screening battery designed for older adults aiming to detect early cognitive changes, particularly in individuals with hearing loss	Performance Index = Reaction time / Correct responses. Higher values indicate worse performance	(Völter et al., 2017)
Cambridge Neuropsychological Test Automated Battery (CANTAB)	Attention, memory (visual and working), executive function, language, visuospatial skills, processing speed	Computerized battery of neuropsychological tests designed to assess a broad range of cognitive functions, widely used in clinical and research settings	Scoring depends on the subtest, including accuracy, latency (ms), and error rates. Results interpreted using age-, sex-, and education-adjusted z-scores.	(Robbins et al., 1994)
CERAD Neuropsychological Battery	Memory (word list learning, recall, recognition), language (naming), visuospatial abilities, executive function	Standardized battery developed to assess and track cognitive decline in older adults, primarily used for diagnosing and evaluating the progression of Alzheimer's disease	Total score range: 0–100. Scores <85 may suggest cognitive impairment.	(Morris et al., 1989)
Cognitive Disorders Examination (CODEX)	Global cognition, memory, orientation, executive function	Brief screening tool designed to rapidly identify cognitive impairment and differentiate between mild cognitive impairment and dementia	Total score: 0–100. Scores <78 suggest Mild Cognitive Impairment (MCI); <67 indicates probable dementia. Higher scores = better cognitive function.	(Larner, 2013)
Cogstate Brief Battery	Processing speed, attention, working memory, learning, memory, and executive function	Short, computerized cognitive assessment designed to quickly measure key cognitive abilities, for both clinical and research use	Provides measures of accuracy (% correct), response time (ms), and composite z-scores. Lower scores indicate poorer performance	(Maruff et al., 2013)
Montreal Cognitive Assessment (MoCA)	Visuospatial and executive function, naming, memory, attention, language, abstraction, and orientation	General cognitive screening tool for detecting mild cognitive impairment and early dementia	Total score: 0–30. Scores <26 typically suggest MCI. Add 1 point for ≤12 years of education. Higher scores = better cognitive function	(Nasreddine et al., 2005)
NIH Toolbox Cognition Battery	Executive function, attention, processing speed, episodic memory, language, working memory	Comprehensive set of standardized, computerized assessments designed to measure key cognitive functions across the lifespan, enabling benchmarking and tracking of cognitive changes in clinical and research settings	T-score system, with a mean of 50 and a standard deviation of 10. Higher scores = better cognitive function	(Weintraub et al., 2013)
Mini-Mental State Examination (MMSE)	Orientation, registration, attention/calculation, recall, and language	General screening tool for detecting dementia.	Total score: 0–30 Scores < 24 suggests cognitive impairment: 19–23 = mild, 10–18 = moderate, < 10 = severe impairment.	(Folstein et al., 1975)
Self-Administered Gerocognitive Examination (SAGE)	Orientation, language, reasoning, visuospatial	Self-administered screening tool designed to detect early signs of cognitive impairment and dementia	Total score: 0–22 Scores < 17 indicates MCI, < 15 suggests dementia	(Scharre et al., 2010)

	abilities, executive function, memory			
Repeatable Battery for the Assessment of Neuropsychological Status (RBANS)	Immediate memory, visuospatial/constructional abilities, language, attention, delayed memory	Brief, standardized assessment of multiple cognitive domains to detect and track neurocognitive decline	Total score: 0 -100<br>Scores < 85 suggests impairment; < 70 indicates severe impairment	(Randolph et al., 1998)
Memory				
5-Word Test	Episodic memory (immediate and delayed recall)	Brief, sensitive screening tool designed to detect early memory impairments, particularly for identifying Mild Cognitive Impairment (MCI) and early Alzheimer's disease	Scoring range: 0–10<br>Recall < 5 suggests MCI or early Alzheimer's	(Dubois et al., 2002)
Brief Visuospatial Memory Test (BVMT)	Visual learning, immediate recall, delayed recall, recognition memory	Measures the ability to learn, recall, and recognize visual stimuli over multiple trials	Total recall score: 0–36<br>Delayed recall score: 0–12.<br>Higher scores = better cognitive performance	(Benedict et al., 1996)
California Verbal Learning Test–II (CVLT-II*)*	Verbal learning, immediate recall, delayed recall, recognition memory, semantic clustering, interference	Assesses verbal memory processes including encoding, retrieval, and recognition, with an emphasis on learning strategies and error patterns	Total raw score: 0-80<br>Lower performance = encoding/retrieval issues	(Delis et al., 2000)
Hopkins Verbal Learning Test–Revised (HVLT-R)	Verbal learning, immediate recall, delayed recall, recognition memory	Assesses verbal memory, including learning rate, retention, and retrieval through recall and recognition tasks	Total recall score : 0–36<br>Delayed score: 0–12<br>Recognition score : 0–12.<br>Higher scores = better memory	(Brandt & Benedict, 2001)
Non-Verbal Learning Test (NVLT)	Nonverbal learning, visual cognition, reasoning, memory	Evaluates the ability to learn, recall, and solve problems using nonverbal stimuli (e.g., shapes, designs)	Scores reflect correct and false positive answers; their difference is also recorded<br>Lower scores = learning disability or other cognitive impairment.	(Sturm & Willmes, 1999)
Picture Sequence Memory Test	Episodic memory, visual memory, sequencing	Assesses the ability to remember and correctly sequence a series of illustrated events or pictures, evaluating temporal order memory and learning across trials	Scored based on the number of correctly recalled adjacent pairs of pictures.<br>lower scores = reduced episodic memory	(Dikmen et al., 2014)
Rey Auditory Verbal Learning Test (RAVLT)	Verbal learning, immediate memory, delayed recall, recognition memory	Assesses the ability to encode, consolidate, store, and retrieve verbal information across multiple learning and recall trials	Total Recall score: 0-75<br>Delayed score: 0–15<br>Higher scores = better memory	(Rey, 1958)
Executive Function				
Auditory Stroop Task	Selective attention, inhibitory control, auditory processing	Measures the capacity to inhibit automatic responses to conflicting auditory cues by requiring participants to respond to one	Scoring: Accuracy (%) and response time (ms)	(Morgan & Brandt, 1989)

		aspect of a stimulus (e.g., word meaning) while ignoring another (e.g., pitch)	High interference score or longer reaction time = impaired inhibition	
Categorization Working Memory Task (CWMT)	Working memory, executive function, categorization	Assesses the ability to hold, update, and manipulate categorized information in working memory while engaging in rule-based sorting	Scoring: Accuracy (%) and response time (ms)<br>Poor accuracy or slow response suggests impairment.	(Conway et al., 2005)
Digit Span	Working memory (forward and backward span), attention, auditory-verbal short-term memory	Evaluates the capacity to maintain and manipulate auditory information in immediate memory	Forward score: 0 - 16<br>Backward score: 0-14<br><br>Forward higher score = higher memory span or capacity<br>Backward higher score = higher mental manipulation or working memory	(Wechsler, 2008)
Digit Symbol Coding	Processing speed, attention, visual-motor coordination, working memory	Evaluates the speed and accuracy of processing by requiring participants to match symbols to numbers using a key, within a limited time	Score: 0–133<br>Lower scores indicate reduced processing speed and cognitive decline	(Wechsler, 2008)
Go/No-Go Test	Inhibitory control, sustained attention, executive function	Evaluates the ability to execute or withhold a motor response depending on presented cues, often used to evaluate impulsivity and self-regulation	Score: Error count and reaction time (ms)<br>High commission errors = poor inhibitory control.	(Luria, 1966)
Hayling Sentence Completion Test	Executive function, response initiation, response inhibition, verbal processing	Evaluates the ability to generate appropriate or inhibited responses by completing sentences with either logical or unrelated words, measuring both initiation speed and inhibitory control	Score: 1 - 10<br>Response time; error count<br><br>Lower scores = impaired cognitive function	(Burgess & Shallice, 1997)
Letter-Number Sequencing Task	Working memory, attention, executive function	Evaluates the ability to simultaneously process and manipulate mixed verbal and numerical information	Scoring range: 0–21<br>Lower scores = working memory deficits	(Wechsler, 2008)
N-Back Test	Working memory, attention, executive function	Measures the capacity to hold and manipulate information over short periods by requiring continuous updating and response to stimuli presented n steps earlier	Scoring: Accuracy (%) and response time (ms)<br>Lower accuracy = poorer working memory performance	(Gevins & Cutillo, 1993)
Raven's Progressive Matrices	Abstract reasoning, fluid intelligence, problem-solving, nonverbal reasoning	Assesses the ability to identify patterns and solve problems using visual stimuli, widely used to evaluate nonverbal reasoning and general intelligence	Score: # of correct answers and response time<br>Fewer correct responses = reduced abstract reasoning.<br>Shorter response time = better processing speed	(Raven, 2003)
Stroop Word Color Test	Attention, processing speed, cognitive inhibition, executive function	Assesses the ability to inhibit automatic responses and manage cognitive interference	Score: correct responses and response time (ms)	(Stroop, 1935)

		by requiring participants to name the ink color of incongruent color words	High interference effect = reduced inhibitory control	
Symbol Search	Processing speed, visual perception, attention, visual scanning	Assesses the speed and accuracy of visual processing by requiring individuals to quickly identify whether target symbols appear among a set of distractors	Scoring: Total correct responses in fixed time<br>Fewer corrections = slower visual processing	(Wechsler, 2008)
Symbol Span	Visual working memory, attention, sequential processing	Assesses the ability to encode, maintain, and recall sequences of abstract visual symbols in the correct order	Score: 1-19<br>Lower scores = deficits in visual working memory and sequencing	(Wechsler, 2008)
Trail Making Test (TMT)	Processing speed (Part A), attention, visual scanning, cognitive flexibility, executive function (Part B)	Assesses visual attention, task switching, and mental flexibility by requiring individuals to connect sequences of numbers (Part A) and alternating numbers and letters (Part B)	Score: response time (ms)<br>maximum time limit, 180 seconds for Part A and 300 seconds for Part B<br>Longer completion time= higher impaired flexibility and executive function	(Reitan, 1958)
Timed up and go (TUG)	Executive function, global cognition, balance	Records the time (in seconds) it takes an individual to rise from a chair, walk a distance of three meters (i.e 10 feet), turn, return to the chair, and sit down again.	Score: response time (ms)<br>Longer completion time = increased risk of future dementia occurrence	(Podsiadlo and Richardson, 1991)
Attention				
d2 Test of Attention	Selective attention, sustained attention, processing speed, visual scanning	Assesses the ability to focus and maintain attention over time by requiring individuals to identify target characters among visually similar distractors under time pressure	Score: total number of items processed (speed), errors (accuracy), and a combined score of speed and accuracy.<br>Higher score = better overall attentional performance	(Brickenkamp & Zillmer, 1998)
Multiple Features Target Cancellation	Selective attention, visual search, processing speed, inhibitory control	Measures the ability to detect and respond only to targets with specific combinations of features while ignoring distractors, requiring efficient scanning and inhibition of irrelevant stimuli	Score: Accuracy and completion time (ms)<br>Low accuracy/longer time = selective attention deficit.	(Mirsky et al., 1991)
Letter Detection Test	Selective attention, visual scanning, processing speed	Evaluates the ability to detect specific letters (or letter combinations) within text or arrays	Score: Total characters processed minus total errors<br>Low performance = attention/concentration issues	(Zygouris & Tsolaki, 2015)
Language				
Boston Naming Test (BNT*)*	Language, specifically confrontational word retrieval and lexical access	Measures naming ability by requiring individuals to name pictured objects of increasing difficulty, useful for detecting anomia and language impairments	Score: 0–60<br>Scoring < 45 suggests anomia or naming impairment.	(Kaplan et al., 2001)

Cardebat's fluency	Language (lexical retrieval), executive function (verbal initiation, flexibility)	Assesses verbal fluency by evaluating both phonemic (letter-based) and semantic (category-based) retrieval	Score: # of words in 60s<br>Scoring < 12 phonemic or < 15 semantic = lexical or executive deficit	(Cardebat et al., 1990)
Controlled Oral Word Association Tests	Verbal fluency, executive function (initiating, switching)	Evaluates phonemic (letter-based) fluency by requiring participants to generate words beginning with specific letters under time limits	Score: # of words per minute<br>Scoring < 12 suggests reduced phonemic fluency.	(Benton et al., 1976)
Test de Dénomination Orale d'images **(**DO80)	Language (naming, lexical retrieval)	Assesses confrontation naming abilities in French-speaking individuals by asking them to name a series of 80 images, helping to detect anomia or other language production impairments	Score: 0–80 correct<br>Scoring < 70 indicates naming difficulty.	(Deloche & Hannequin, 1997)
Phonemic and semantic fluency tasks	Language, lexical access	Evaluates verbal fluency by requiring individuals to generate as many words as possible either beginning with a specific letter (phonemic) or belonging to a category (semantic) within a limited time	Score: # of words per minute<br>Lower output = impaired lexical access	(Lezak et al., 2012)
Regensburg Word Test (RWT)	Verbal fluency, lexical retrieval, executive function	Assesses phonemic, semantic, and alternate forms of word fluency in German	Score: # words per category/letter<br>Lower score = lexical retrieval or executive dysfunction.	(Aschenbrenner et al., 2000)
Visuospatial abilities				
Clock Drawing Test (CDT)	Visuospatial abilities, executive function, attention, planning, numerical knowledge, motor control	Assesses multiple cognitive functions by requiring individuals to draw a clock with a specified time, commonly used as a quick screening tool for cognitive impairment and dementia	Score: 0–10<br>Scores < 8 = visuospatial/executive dysfunction.	(Shulman, 2000)
Corsi Block-tapping Test	Visuospatial short-term memory, working memory	Evaluates the capacity to recall and reproduce sequences of tapped blocks	Score: 0-36<br>Forward score: 0-16<br>Backward score: 0-16<br><br>Higher scores = higher spatial capacity	(Corsi, 1973)
Groton Maze Learning Test (GMLT	Spatial working memory, learning efficiency, executive function, visuospatial processing	Estimates the ability to learn and recall a hidden pathway through a maze over repeated trials, measuring error monitoring, strategy use, and cognitive flexibility	Score: Errors and completion time (ms)<br>More errors = poor planning and spatial learning	(Pietrzak et al., 2009)
Rey-Osterrieth Complex Figure Test	Visuospatial construction, visual memory, planning, organization, attention, executive function	Assesses visuospatial abilities and nonverbal memory by having individuals copy and later recall a complex geometric figure, evaluating both accuracy and strategy	Score: 0–36 for copy & recall scores<br>Copy < 20 or recall < 15 = visual/spatial impairment.	(Osterrieth, 1944)

Spatial span	Visuospatial working memory, attention	Measures the capacity to encode, maintain, and recall spatial information by requiring individuals to reproduce progressively longer sequences of tapped or pointed locations	Score: 0-36<br>Forward sore 0-16<br>Backward score 0-16<br><br>Higher scores = higher spatial capacity	(Wechsler, 2008)
Reading skills				
National Adult Reading Test–Revised (NART-R)	Word reading ability, estimation of premorbid intelligence	Provides an estimate of premorbid verbal IQ by assessing the correct pronunciation of irregular words, commonly used for evaluating baseline cognitive function in clinical or research settings	Score: # of correct pronunciations<br>Lower scores = lower baseline ability.	(Nelson & Willison, 1991)
Test of Premorbid Functioning (TOPF)	Premorbid intelligence, reading ability	Estimates an individual's pre-illness (premorbid) cognitive functioning level—often used to assess the degree of cognitive decline relative to baseline ability	Score: pronunciation and demographics; lower scores = lower estimated premorbid function	(Wechsler, 2011)
Test of Word Reading Efficiency (TOWRE)	Reading fluency, decoding, sight word recognition, phonemic decoding efficiency	Measures the ability to accurately and rapidly decode printed words, including both real words and pseudowords, providing an index of reading efficiency	Score: # of pronounced printed words and phonemically regular nonwords accurately and fluently.<br>lower scores = poorer reading fluency	(Torgeson et al., 1999)
Wide Range Achievement Test (WRAT)	Reading, spelling, arithmetic skills	Assesses fundamental academic skills to determine achievement levels in reading, spelling, and math for diagnostic, placement, or research purposes	Reading Composite Raw Score = Word Reading Standard Score + Sentence Comprehension Standard Score<br>Lower score = academic skill deficits	(Wilkinson & Robertson, 1993)

References

Aschenbrenner, S., Tucha, O., & Lange, K. W. (2000). *RWT: Regensburger Wortflüssigkeits-Test*. Hogrefe.

Benedict, R. H. B., Schretlen, D., Groninger, L., Dobraski, M., & Shpritz, B{Citation}. (1996). Revision of the Brief Visuospatial Memory Test: Studies of normal performance, reliability, and validity. *Psychological Assessment*, *8*(2), 145–153. https://doi.org/10.1037/1040-3590.8.2.145

Benton, A., Hamsher, K., & Sivan, A. (1976). *Multilingual Aphasia Examination (Iowa City, IA: University of Iowa)*.

Brandt, J., & Benedict, R. H. B. (2001). *Hopkins Verbal Learning Test–Revised: Professional manual*. Psychological Assessment Resources.

Brickenkamp, R., & Zillmer, E. (1998). *The d2 Test of Attention*. Hogrefe & Huber Publishers.

Burgess, P. W., & Shallice, T. (1997). *The Hayling and Brixton Tests*. Thames Valley Test Company.

Cardebat, D., Doyon, B., Puel, M., Goulet, P., & Joanette, Y. (1990). Formal and semantic lexical evocation in normal subjects. Performance and dynamics of production as a function of sex, age and educational level. *Acta Neurologica Belgica*, *90*(4), 207–217.

Conway, A. R. A., Kane, M. J., Bunting, M. F., Hambrick, D. Z., Wilhelm, O., & Engle, R. W. (2005). Working memory span tasks: A methodological review and user's guide. *Psychonomic Bulletin & Review*, *12*(5), 769–786. https://doi.org/10.3758/BF03196772

Corsi, P. M. (1973). *Human memory and the medial temporal region of the brain* (Vol. 34, Issues 2-B, p. 891). ProQuest Information & Learning.

Delis, D. C., Kramer, J. H., Kaplan, E., & Ober, B. A. (2000). *California Verbal Learning Test – Second Edition (CVLT-II) Manual*. The Psychological Corporation.

Deloche, G., & Hannequin, D. (1997). *Test de dénomination orale d'images: DO 80*. Éditions du centre de psychologie appliquée.

Dikmen, S. S., Bauer, P. J., Weintraub, S., Mungas, D., Slotkin, J., Beaumont, J. L., & Heaton, R. K. (2014). Measuring episodic memory across the lifespan: NIH Toolbox Picture Sequence Memory Test. *Journal of the International Neuropsychological Society*, *20*(6), 611–619. https://doi.org/10.1017/S1355617714000460

Dubois, B., Touchon, J., Portet, F., Ousset, P. J., Vellas, B., & Michel, B. (2002). ["The 5 words": A simple and sensitive test for the diagnosis of Alzheimer's disease]. *Presse Medicale (Paris, France: 1983)*, *31*(36), 1696–1699.

Folstein, M. F., Folstein, S. E., & McHugh, P. R. (1975). "Mini-mental state": A practical method for grading the cognitive state of patients for the clinician. *Journal of Psychiatric Research*, *12*(3), 189–198. https://doi.org/10.1016/0022-3956(75)90026-6

Gevins, A., & Cutillo, B. (1993). Spatiotemporal dynamics of component processes in human working memory. *Electroencephalography and Clinical Neurophysiology*, *87*(3), 128–143.

Kaplan, E., Goodglass, H., & Weintraub, S. (2001). *Boston Naming Test* (2nd ed.). Pro-Ed.

Larner, A. J. (2013). Codex (cognitive disorders examination) for the detection of dementia and mild cognitive impairment. *La Presse Médicale*, *42*(12), e425–e428. https://doi.org/10.1016/j.lpm.2013.07.014

Lezak, M. D., Howieson, D. B., Bigler, E. D., & Tranel, D. (2012). *Neuropsychological Assessment* (5th ed.). Oxford University Press.

Luria, A. R. (1966). *Higher cortical functions in man*. Basic Books.

Maruff, P., Lim, Y. Y., Darby, D., Ellis, K. A., Pietrzak, R. H., Snyder, P. J., Bush, A. I., Szoeke, C., Schembri, A., Ames, D., Masters, C. L., & for the AIBL Research Group. (2013). Clinical utility of the cogstate brief battery in identifying cognitive impairment in mild cognitive impairment and Alzheimer's disease. *BMC Psychology*, *1*(1), 30. https://doi.org/10.1186/2050-7283-1-30

Mirsky, A. F., Anthony, B. J., Duncan, C. C., Ahearn, M. B., & Kellam, S. G. (1991). Analysis of the elements of attention: A neuropsychological approach. *Neuropsychology Review*, *2*(2), 109–145. https://doi.org/10.1007/BF01109051

Morgan, A. L., & Brandt, J. F. (1989). An auditory Stroop effect for pitch, loudness, and time. *Brain and Language*, *36*(4), 592–603. https://doi.org/10.1016/0093-934x(89)90088-6

Morris, J. C., Heyman, A., Mohs, R. C., Hughes, J. P., van Belle, G., Fillenbaum, G., Mellits, E. D., & Clark, C. (1989). The Consortium to Establish a Registry for Alzheimer's Disease (CERAD). Part I. Clinical and neuropsychological assessment of Alzheimer's disease. *Neurology*, *39*(9), 1159–1165. https://doi.org/10.1212/wnl.39.9.1159

Nasreddine, Z. S., Phillips, N. A., Bédirian, V., Charbonneau, S., Whitehead, V., Collin, I., Cummings, J. L., & Chertkow, H. (2005). The Montreal Cognitive Assessment, MoCA: A Brief Screening Tool For Mild Cognitive Impairment. *Journal of the American Geriatrics Society*, *53*(4), 695–699. https://doi.org/10.1111/j.1532-5415.2005.53221.x

Nelson, H. E., & Willison, J. (1991). *National adult reading test (NART).* Nfer-Nelson Windsor.

Osterrieth, P.-A. (1944). Le test de copie d'une figure complexe: Contribution à l'étude de la perception et de la mémoire. *Archives de Psychologie*, *30*, 206–356.

Pietrzak, R. H., Snyder, P. J., Jackson, C. E., & Maruff, P. (2009). Characterizing cognitive dysfunction in carriers of the Huntington's disease mutation in the prediagnosis phase: An application of the Cogstate C3 battery. *Archives of Clinical Neuropsychology*, *24*(7), 689–698. https://doi.org/10.1093/arclin/acp064

Podsiadlo, D., and Richardson, S. (1991). The timed "up & go": a test of basic functional mobility for frail elderly persons. J. Am. Geriatr. Soc. 39, 142–148. doi: 10.1111/j.1532-5415.1991.tb01616.x

Randolph, C., Tierney, M. C., Mohr, E., & Chase, T. N. (1998). The Repeatable Battery for the Assessment of Neuropsychological Status (RBANS): Preliminary clinical validity. *Journal of Clinical and Experimental Neuropsychology*, *20*(3), 310–319. https://doi.org/10.1076/jcen.20.3.310.823

Raven, J. (2003). Raven progressive matrices. In *Handbook of nonverbal assessment* (pp. 223–237). Springer.

Reitan, R. M. (1958). Validity of the Trail Making Test as an indicator of organic brain damage. *Perceptual and Motor Skills*, *8*(3), 271–276. https://doi.org/10.2466/pms.1958.8.3.271

Rey, A. (1958). *L'examen clinique en psychologie.*

Robbins, T. W., James, M., Owen, A. M., Sahakian, B. J., McInnes, L., & Rabbitt, P. M. A. (1994). Cambridge Neuropsychological Test Automated Battery (CANTAB): A factor analytic study of a large sample of normal elderly volunteers. *Dementia*, *5*(5), 266–281. https://doi.org/10.1159/000106735

Scharre, D. W., Chang, S. I., Murden, R. A., Lamb, J., Beversdorf, D. Q., Kataki, M., Nagaraja, H. N., & Bornstein, R. A. (2010). Self-administered gerocognitive examination (SAGE): A brief cognitive assessment instrument for mild cognitive impairment (MCI) and early dementia. *Alzheimer Disease and Associated Disorders*, *24*(1), 64–71. https://doi.org/10.1097/WAD.0b013e3181b03277

Shulman, K. I. (2000). Clock-drawing: Is it the ideal cognitive screening test? *International Journal of Geriatric Psychiatry*, *15*(6), 548–561. https://doi.org/10.1002/1099-1166(200006)15:6<548::AID-GPS242>3.0.CO;2-U

Stroop, J. R. (1935). Studies of interference in serial verbal reactions. *Journal of Experimental Psychology*, *18*(6), 643–662. https://doi.org/10.1037/h0054651

Sturm, W., & Willmes, K. (1999). *Nonverbaler Lerntest (NVLT)*. Hogrefe.

Torgeson, J. K., Wagner, R. K., & Rashotte, C. A. (1999). Test review: Test of word reading efficiency (TOWRE). *Inc.: Austin, TX, USA*.

Völter, C., Götze, L., Falkenstein, M., Dazert, S., & Thomas, J. P. (2017). Application of a computer-based neurocognitive assessment battery in the elderly with and without hearing loss. *Clinical Interventions in Aging*, *12*, 1681–1690. https://doi.org/10.2147/CIA.S142541

Wechsler, D. (2008). *Wechsler Adult Intelligence Scale—Fourth Edition* [Dataset]. https://doi.org/10.1037/t15169-000

Wechsler, D. (2011). The Test of Premorbid Functioning (TOPF) The Psychological Corporation. *San Antonio, TX, USA*.

Weintraub, S., Dikmen, S. S., Heaton, R. K., Tulsky, D. S., Zelazo, P. D., Bauer, P. J., Carlozzi, N. E., Slotkin, J., Blitz, D., Wallner-Allen, K., Fox, N. A., Beaumont, J. L., Mungas, D., Nowinski, C. J., Richler, J., Deocampo, J. A., Anderson, J. E., Manly, J. J., Borosh, B., … Gershon, R. C. (2013). Cognition assessment using the NIH Toolbox. *Neurology*, *80*(11 Suppl 3), S54–S64. https://doi.org/10.1212/WNL.0b013e3182872ded

Wilkinson, G. S., & Robertson, G. J. (1993). Wide range achievement test 4. *Journal of Clinical and Experimental Neuropsychology*.

Zygouris, S., & Tsolaki, M. (2015). Computerized Cognitive Testing for Older Adults. *American Journal of Alzheimer's Disease and Other Dementias*, *30*(1), 13–28. https://doi.org/10.1177/1533317514522852
