# Supplementary figures and images for: Neuropsychological Assessments to Explore the Cognitive Impact of Cochlear Implants: A Scoping Review
Source: J Clin Med. 2025 Oct 27;14(21):7628. doi: 10.3390/jcm14217628 (PMC12608580; doi:10.3390/jcm14217628)

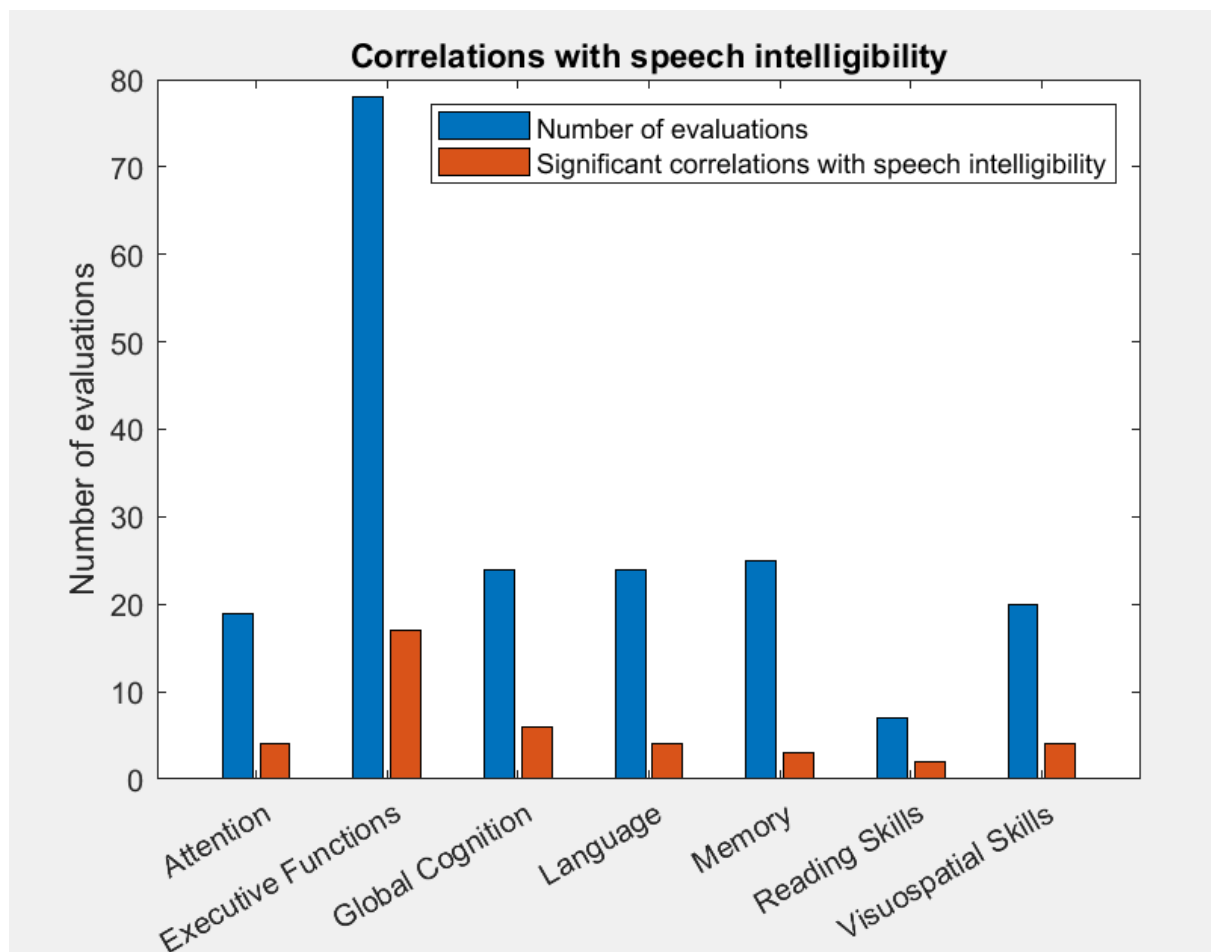

**Figure S3.** Correlations between speech intelligibility and cognitive domain.

Supplement: Supplementary file 1 [file jcm-14-07628-s001.zip › Figure S3. Correlations between speech intelligibility and cognitive domain. .pdf]
